# GC-MS metabolomics reveals potential biomarkers for infection-specific subtypes in sepsis

**DOI:** 10.1515/med-2026-1539

**Published:** 2026-09-24

**Authors:** Cheng Chen, Xinna Zhao

**Affiliations:** Department of Emergency and Critical Care Medicine, Shanghai General Hospital Affiliated to Shanghai Jiao Tong University School of Medicine, Shanghai, China; Department of Cardiology, Shanghai Tongji Hospital, Tongji University School of Medicine, Shanghai, China

**Keywords:** sepsis, candidate biomarker, metabolite, pulmonary infection, abdominal infection, urinary tract infection

## Abstract

**Objectives:**

Sepsis remains one of the most severe complications of infection. Specific and easily measurable biomarkers could help us predict the type of infection in sepsis. This study aimed to explore biomarkers for early identification of sepsis with different infection.

**Methods:**

Serum was obtained from 37 patients with sepsis and 8 healthy individuals. Metabolites were screened using gas chromatography-mass spectrometry (GC-MS), and then potential biomarkers were screened. Receiver operating characteristic curve (ROC) analysis was used to evaluate potential biomarkers.

**Results:**

A total of 24 differential metabolites were found between sepsis subgroup with pulmonary infection and healthy controls, and they were enriched with 8 metabolic pathways. Similarly, 27 differential metabolites were identified in abdominal infection cases, and 64 in urinary tract infection (UTI) cases, which were associated with 23 and 26 metabolic pathways, respectively. Hydroxylamine and N-acetylputrescine may be used as candidate biomarkers to distinguish pulmonary infection and abdominal infection in sepsis, respectively, while glucose, phosphoenolpyruvate and d-ribose were good discriminators in UTI.

**Conclusions:**

Our GC-MS-based metabolomic study identified potential biomarkers for sepsis across various infection types. These biomarkers show promise for the rapid and precise diagnosis of sepsis onset. Our GC-MS-based metabolomic study identified candidate metabolites associated with different infection sources in sepsis. Given the limited sample size and the correspondingly limited statistical power, these findings are exploratory and require prospective validation in an adequately powered, independent cohort before any diagnostic application can be considered.

## Introduction

Sepsis, defined by life-threatening circulatory, cellular, and metabolic dysfunction, remains a major cause of high mortality and a severe complication of infection. It is characterized by a dysregulated host response to infection that can lead to tissue and organ damage [1]. As a resource-intensive condition, sepsis requires prompt recognition and management to improve patient outcomes. Consequently, there is a critical need for biomarkers that can enable rapid, accurate diagnosis [2].

Imaging modalities are widely used to localize infection site in sepsis. CT offers high resolution but involves ionizing radiation, limiting its repeated use in pregnant women and children. Ultrasound is radiation-free, bedside-accessible, and useful for abdominal and urinary tract sources, though it is operator-dependent and limited for pulmonary or deep-seated infection. MRI provides excellent soft-tissue contrast without radiation but is time-consuming, costly, and impractical for unstable critically ill patients [3], 4]. Despite these complementary tools, none provides a rapid molecular biomarker of sepsis onset or infection type, underscoring the need for easily measurable biomarkers. Numerous laboratory tests are employed in sepsis diagnosis, including complete blood count, platelet count, and C-reactive protein(CRP) levels. Studies indicate that procalcitonin(PCT) serves as a valuable biomarker for detecting sepsis in critically ill patients, and its accuracy and clinical utility have been well validated [5], 6]. Nevertheless, a specific test for the onset of sepsis remains unavailable. While blood culture analysis is a reliable method for confirming the causative pathogen in sepsis, its diagnostic value is constrained by low sensitivity and frequent contamination [7]. Next-generation sequencing can identify pathogens responsible for infections but is costly and time-consuming. To date, no biomarker has been established for the rapid and effective prediction of sepsis onset. Identifying specific and easily measurable biomarkers could aid in predicting the type of infection in sepsis.

Recent studies have highlighted the value of metabolic profiling in the identification of sepsis, owing to its capacity to detect distinct metabolite patterns. Metabolites represent the end products of interactions between pathogens and host physiological, genetic, and environmental factors; therefore, metabolic profiles can reflect the individual functional status [8]. In this study, we aimed to investigate metabolic characteristics and identify candidate biomarkers that may help characterize and classify the infection source in patients already diagnosed with sepsis using gas chromatography-mass spectrometry (GC-MS).

## Materials and methods

### Study population

This study enrolled patients with sepsis from the Department of Emergency and Critical Care at Shanghai General Hospital affiliated to Shanghai Jiao Tong University School of Medicine between June 2020 and October 2021, with diagnosis based on the SEPSIS 3.0 criteria. All patient management adhered to the contemporary Surviving Sepsis Campaign guidelines. All patients were screened and enrolled within 24 h after being diagnosed with sepsis, and sample collection was completed within 24 h after being diagnosed. Patients were excluded if they have (1) Currently suffering from active autoimmune diseases, Corticosteroid (prednisone or equivalent drug dose>10 mg/d), (2)the immunosuppressive therapy should be received within 14 days before enrollment, (3)active stage of malignant tumor, (4) Pregnancy or lactation period. A control group of healthy volunteers was established from the physical examination center of Shanghai General Hospital, consisting of individuals with normal test results and no remarkable medical history. Relevant clinical and demographic data are detailed in the results.

### Sample preparation

All samples were collected within 24 h after the patient was diagnosed with sepsis. Serum was separated within 30 min in 4 °C of collection by centrifuging at 2,200 rpm for 10 min, and stored at −80 °C; the storage duration before analysis was 3 months. Samples underwent a single freeze-thaw cycle and were thawed on ice (4 °C) immediately before preparation. The sample preparation procedure was as our previous study [8]. 20 μL of 2-chloro-l-phenylalanine in methanol (internal standard) was added to 80 μL of thawed serum. Then, 240 μL of methanol:acetonitrile (2:1, v/v) was introduced to the mixture. The mixture was subjected to extraction by ultrasonication for 8 min in an ice-water bath, followed by centrifugation at 12,000 rpm for 10 min at 4 °C. A 150 μL of the aliquot supernatant was transferred and dried under vacuum at 24 °C. The residue was treated with 80 μL of methoxylamine, vortexed, and incubated at 37 °C for 90 min. Subsequently, 50 μL of BSTFA (with 1 % TMCS) and 20 μL of n-hexane were added. The mixture vortexed and reacted at 70 °C for 60 min, and then equilibrated at 24 °C for 30 min before GC-MS analysis. Quality control (QC) samples (pooled aliquots) were injected at regular intervals throughout the run to monitor analytical stability; metabolites with QC RSD >30 % were excluded.

### Analytical and metabolomics profiling

An untargeted metabolomic profiling was conducted using an Agilent 7890B-5977B GC-MS system (Agilent, Palo Alto, CA). Chromatographic separation was achieved on a 30 m × 0.25 mm × 0.25 μm HP-5MS fused-silica capillary column (Agilent J & W Scientific, CA) with helium (>99.999 %) as the carrier gas at a constant flow rate of 1 mL/min. The injector temperature was set at 260 °C. The mass spectrometer was operated under the following conditions: ion source temperature, 230 °C; quadrupole temperature, 150 °C; and electron impact energy, 70 eV. Data were acquired in full-scan mode across a mass range of m/z 50–500. All samples were analyzed in random order with integrated quality control measures. The data matrix was imported into R for principal component analysis (PCA) to assess overall sample distribution and analytical stability. Furthermore, partial least squares discriminant analysis (PLS-DA) and orthogonal PLS-DA (OPLS-DA) were applied via SIMCA 14.1 (Umetrics, Sweden) to visualize inter-group metabolic differences. Metabolite identification was carried out by querying the National Institute of Standards and Technology database, and pathway enrichment analysis was based on the Kyoto Encyclopedia of Genes and Genomes (KEGG) database. Variable importance of projection (VIP) values obtained from the OPLS-DA model were used to rank the overall contribution of each variable to group discrimination. A two-tailed Student’s T-test was further used to verify whether the metabolites of difference between groups were significant. Differential metabolites were selected with VIP values greater than 1.0 and p-values <0.05.

Metabolite levels were determined by relative quantification. Peak areas of each metabolite were normalized to that of the single internal standard (2-chloro-l-phenylalanine) and are expressed as relative abundances (peak-area ratios). No absolute quantification with authentic external calibration standards or compound-specific stable-isotope-labeled internal standards was performed. Consequently, all inter-group comparisons were based on the relative abundance of the same metabolite across groups rather than on comparisons of absolute concentrations between different metabolites.

### Statistical analysis

Statistical analyses were conducted in GraphPad Prism (v8.2.1). Prior to analysis, all data were assessed for normality to guide the selection of appropriate parametric or nonparametric tests. For clinical data, inter-group comparisons were conducted using the parametric Student’s t-test or the chi-square test, as applicable. In the metabolomics analysis, a two-tailed Student’s t-test was first used to identify metabolites with significant differences between groups. Subsequently, one-way ANOVA was applied to determine the statistical significance of these metabolites. Differential metabolites were defined as those satisfying a VIP value >1.0 and a p-value <0.05. The diagnostic potential of these significant metabolites was further evaluated via ROC curve analysis. A p-value of <0.05 was considered statistically significant for all tests. Because the subgroup sizes were fixed by the available clinical cohort, no *a priori* sample-size calculation was performed; instead, a post hoc power analysis was conducted to define the effect sizes the study was able to detect. For each two-group comparison, the minimum detectable standardized effect size (Cohen’s d) at α=0.05 (two-tailed) and 80 % power was calculated for a two-sample t-test, and the achieved power was estimated from the observed effect size of each candidate biomarker. Power for the ROC analyses was estimated for the null hypothesis AUC=0.5 using the Hanley-McNeil variance approximation at α=0.05. Calculations were performed in GPower 3.1 and R (packages pwr and pROC). Because the metabolite panel was screened without formal correction for multiple comparisons, all p-values are reported as nominal and are interpreted as exploratory rather than confirmatory.

### Ethics and participant informed consent

The medical research study described in this paper was approved by the Ethics Committee of Shanghai General Hospital, Shanghai Jiao Tong University School of Medicine (Approval No. [2019]11). Informed consent was obtained from all individuals included in this study, or their legal guardians or wards.

## Results

### Characteristics of patients and healthy controls

**Figure 1:**
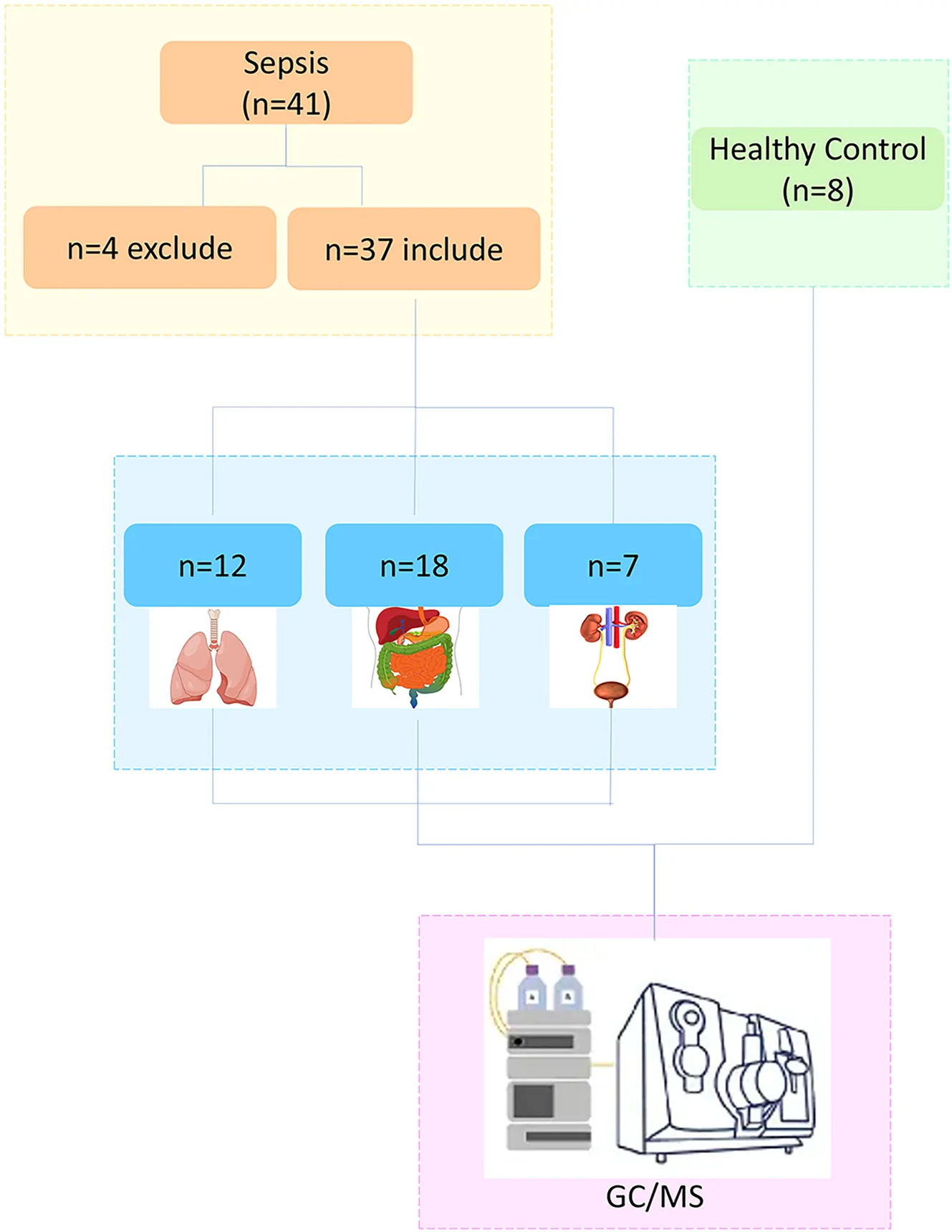
The study cohort. Four sepsis patients were excluded, three patients have malignant tumors and are undergoing radiotherapy and chemotherapy; one patient with autoimmune disease and long-term use of glucocorticoids.

A total of 41 patients meeting Sepsis-3 criteria and 8 healthy volunteers were initially enrolled. Four sepsis patients were excluded, three patients have malignant tumors and are undergoing radiotherapy or chemotherapy; one patient with autoimmune disease and long-term use of glucocorticoids. A total of 37 sepsis patients were enrolled in this study, of which 12 with pulmonary infections, 18 with abdominal infections, and 7 with urinary tract infections(UTI). Alongside 8 healthy controls were recruited (Figure 1). General characteristics and laboratory findings of the patient cohort are detailed in Table 1.

**Table 1:** Demographic and clinical features of patients and healthy controls.

Variables	P	A	U	HC
	(n=12)	(n=18)	(n=7)	(n=8)
Age, years	74 ± 16	70 ± 17	68 ± 8	62 ± 11
Males/females	8/4	16/2	0/7	4/4
**Comorbidities, n (%)**				
Hypertension	2 (16.7 %)	8 (44.4 %)	2 (28.5 %)	–
Diabetes mellitus	4 (33.3 %)	4 (22.2 %)	4 (57.1 %)	–
Coronary heart disease	2 (16.7 %)	4 (22.2 %)	3 (42.9 %)	–
COPD	2 (16.7 %)	3 (16.7 %)	0 (0 %)	–
Chronic kidney disease	2 (16.7 %)	3 (16.7 %)	0 (0 %)	–
**Physical signs and laboratory tests**				
Temp. sampling time, °C	37.5 ± 0.9	37.6 ± 1.3	38.7 ± 1.1	–
Heart rate, bpm	94.1 ± 19.9	102.7 ± 15.6	114.6 ± 24.7	–
C-reactive protein, mg/L	113.1 ± 54.8	193.7 ± 106	291.2 ± 102.4	–
Neutrophil, %	85.7 ± 18.4	89.9 ± 7.8	92.2 ± 2.7	–
Lymphocyte, %	6.2 ± 7.3	5.3 ± 4.9	5.0 ± 2.8	–
Monocyte, %	3.7 ± 1.3	3.5 ± 1.7	2.5 ± 0.8	–
Lymphocyte (109/L)	3.1 ± 8.6	0.5 ± 0.5	0.7 ± 0.5	–
Monocyte (109/L)	0.6 ± 0.2	0.4 ± 0.3	0.4 ± 0.3	–
Hemoglobin, g/L	114 ± 34	107 ± 30.6	119.6 ± 17.6	–
ALB, g/L	31.1 ± 6.5	30.2 ± 6.2	31.5 ± 4.5	–
GLU, mmol/L	7.3 ± 2.3	8.9 ± 2.8	11.5 ± 2.9	–
LAC, mmol/L	3.1 ± 3.6	2.3 ± 0.9	6.2 ± 6.1	–
LDH, U/L	634.8 ± 533.1	650.5 ± 299.1	741.0 ± 189.1	–
ALT, U/L	68.8 ± 104.9	29.4 ± 11.9	106.5 ± 180.6	–
AST, U/L	97.8 ± 206.5	36.8 ± 18.4	89.1 ± 85.2	–
γ-GT, U/L	73.1 ± 41.1	36.7 ± 28.1	36.2 ± 20.2	–

Quantitative data are presented as mean ± SD, the above laboratory tests were conducted within 24 h after the patient was diagnosed with sepsis. P, pulmonary infections; A, abdominal infections; U, urinary tract infections; HC, healthy controls.

### Metabolomic profiling distinguishes sepsis patients from healthy controls

**Figure 2:**
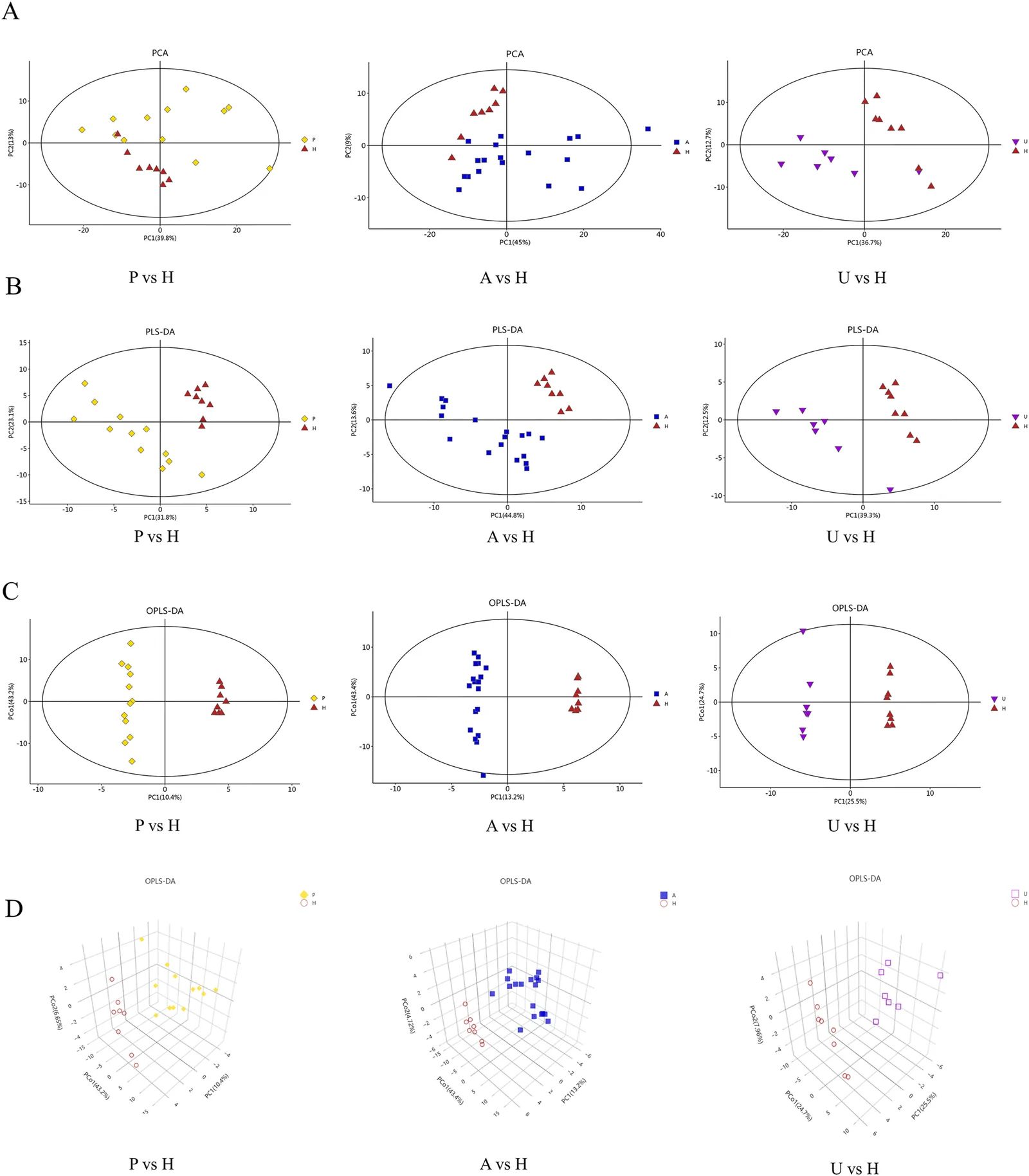
The PCA, PLS-DA, OPLS-DA and 3D OPLS-DA models distinguished patients between groups. A: The red triangles represent heathy controls in the PCA score plots, the yellow squares represent sepsis patients with pulmonary infection, the blue squares represent sepsis patients with abdominal infection, and the purple squares represent sepsis patients with UTI. B: The red triangles represent heathy controls in the PLS-DA score plots, the yellow squares represent sepsis patients with pulmonary infection, the blue squares represent sepsis patients with abdominal infection, and the purple squares represent sepsis patients with UTI. C: The red triangles represent heathy controls in the OPLS-DA score plots, the yellow squares represent sepsis patients with pulmonary infection, the blue squares represent sepsis patients with abdominal infection, and the purple squares represent sepsis patients with UTI. D: The red circle represent heathy controls in the 3D OPLS-DA score plots, the yellow squares represent sepsis patients with pulmonary infection, the blue squares represent sepsis patients with abdominal infection, and the purple squares represent sepsis patients with UTI. Abbreviations: PCA, principle component analysis; PLS-DA, partial least squares discriminant analysis; OPLS-DA, orthogonal projection latent to structure discriminant analysis. P: pulmonary infections; A: abdominal infections; U: urinary tract infections; H: healthy controls.

To investigate metabolic alterations, serum samples from sepsis patients and healthy controls were analyzed by GC-MS. PCA was first employed to assess the overall distribution of samples and the stability of the analytical process (Figure 2A). Subsequently, PLS-DA, a supervised method for identifying inter-group differences, was applied. The PLS-DA model revealed a clear separation between sepsis patients and healthy controls, indicating distinct metabolite profiles (Table2, Figure 2B). To prevent overfitting of the model, seven fold cross validation and 200 response permutation testing (RPT) were used to evaluate the quality of the model. Response ranking test is a random ranking method used to evaluate the accuracy of OPLS models, in order to avoid the accidental classification obtained by supervised learning methods. We present the response ranking test results of OPLS-DA models to enhance model specificity and filter out non-relevant variation. This analysis yielded distinct separations between healthy controls and each subgroup of sepsis (pulmonary infection, abdominal infection, and UTI), as visualized in Figure 2C.

**Table 2:** The quantitative parameters of these models.

Group	Type	PRE	ORT	n	R2X (cum)	R2Y (cum)	Q2 (cum)	R2	Q2
A/H	PCA	3	0	26	0.609				
A/H	PLS	3	0	26	0.615	0.981	0.895		
A/H	OPLS	1	3	26	0.646	0.994	0.877	0.952	−0.417
P/H	PCA	3	0	20	0.6				
P/H	PLS	4	0	20	0.644	0.995	0.906		
P/H	OPLS	1	3	20	0.644	0.995	0.813	0.981	−0.223
U/H	PCA	3	0	15	0.612				
U/H	PLS	3	0	15	0.582	0.997	0.872		
U/H	OPLS	1	2	15	0.582	0.997	0.757	0.961	−0.211

Type: established multivariate statistical analysis model; PRE: represents the number of principal components during modeling; ORT: represents the number of orthogonal components during modeling; n: represents the number of samples during modeling; R2X (cum): represents the cumulative explanatory power of the model in the X-axis direction when modeling multivariate statistical analysis (or can be understood as the square of the percentage of original data information retained in the X-axis direction), and cum represents the cumulative result of several principal components; R2Y (cum): represents the cumulative interpretability of the model in the Y-axis direction (or can be understood as the square of the percentage of original data information retained in the Y-axis direction); Q2 (cum): represents the cumulative prediction rate of the model; R2, Q2: parameters for response ranking test, used to measure whether the model is overfitting. P, pulmonary infections; A, abdominal infections; U, urinary tract infections; HC, healthy controls.

### Differential metabolites were screened by GC-MS

**Figure 3:**
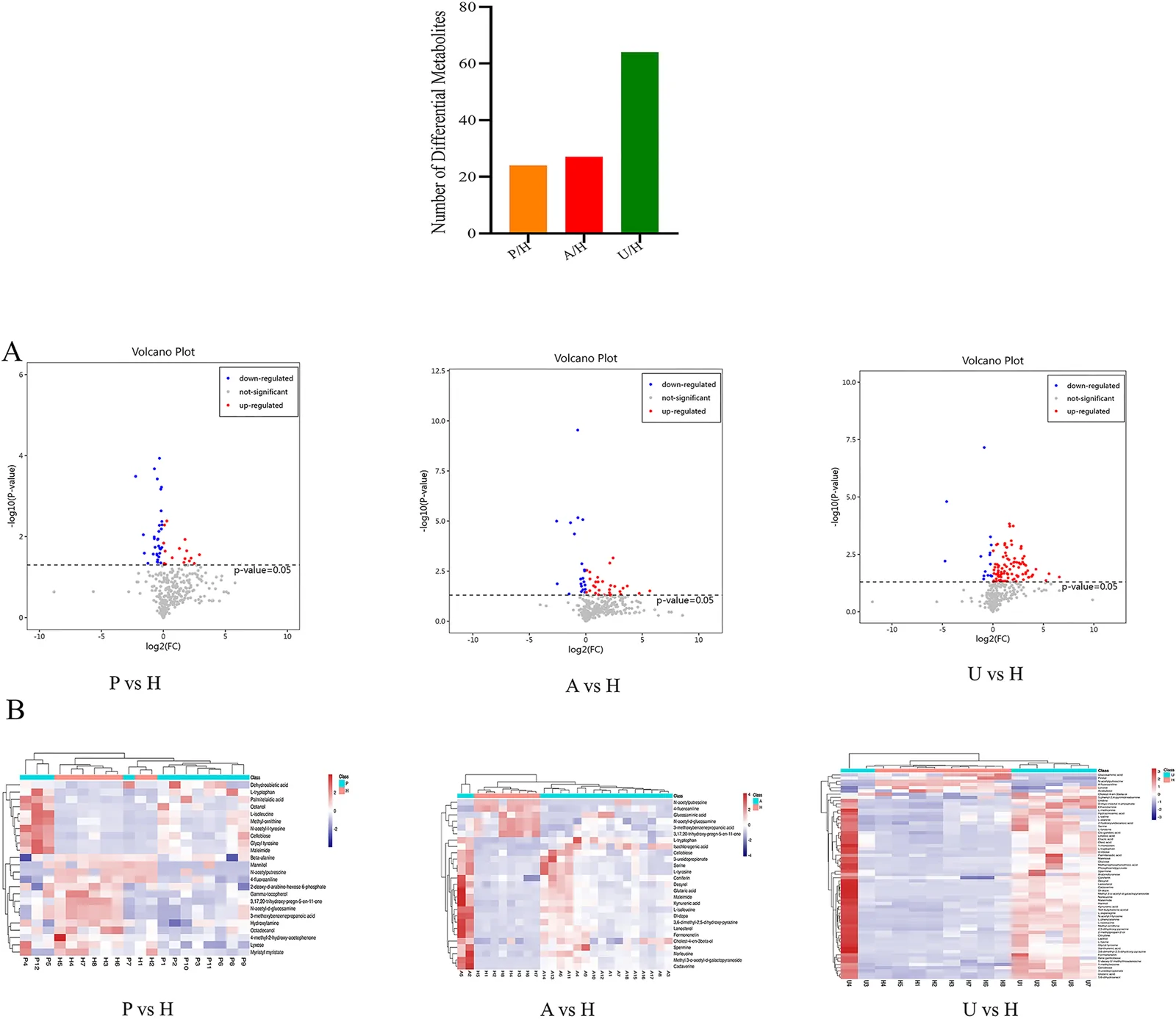
Differential metabolites were detected by GC-MS. A: The histogram showed the quantity of differential metabolites. B: The volcanic map visualized changes in differential metabolites. C: The heatmap demonstrated differential metabolites. P: pulmonary infections; A: abdominal infections; U: urinary tract infections; H: healthy controls.

Differential metabolites were screened from the GC-MS data based on the OPLS-DA model. In this model, the VIP value measures influence of each metabolite and explanatory power in discriminating between groups. Based on these criteria, 24 differential metabolites were found between sepsis subgroup with pulmonary infection and healthy controls, of which 16 were upregulated and 8 were downregulated. Similarly, 27 differential metabolites were identified in abdominal infection cases (20 upregulated), and 64 in UTI cases (58 upregulated). These results are detailed in Table 3 and visualized in Figure 3. The mass spectrometry parameters of each metabolite are shown in Table S1.

**Table 3:** Differential metabolites were screened by GC-MS.

Metabolite name	P vs. H			A vs. H			U vs. H		
	V	VIP	FC	V	VIP	FC	V	VIP	FC
N-acetylputrescine	↓	3.239203619	0.212581115	↓	2.738643672	0.166416507	↓	2.641935206	0.041653338
Mannitol	↓	2.81493208	0.326705467	–	–	–	–	–	–
Glycyl tyrosine	↑	1.958240156	7.510835789	–	–	–	↑	2.053212464	11.90860674
Maleimide	↑	1.899959681	3.690900525	↑	1.84939303	4.268471493	↑	1.727718347	4.42447945
4-Methyl-2-hydroxy-acetophenone	↓	1.872019863	0.346188489	–	–	–	–	–	–
l-isoleucine	↑	1.679633769	4.694669254	↑	1.954351686	5.889528413	↑	1.759370733	6.039506632
Lyxose	↓	1.664446214	0.569153684	–	–	–	↓	1.277936254	0.540149796
Palmitelaidic acid	↑	1.639679104	3.343558175	–	–	–	↑	1.875161307	5.886220218
L-tryptophan	↓	1.55607041	3.355654769	↑	2.131366795	5.33334444	↑	2.070163959	7.693819206
Cellobiose	↓	1.509958829	2.451646576	↑	1.429428466	2.796038398	↑	1.34300124	2.624775846
Octanol	↓	1.45887901	3.155653079	–	–	–	–	–	–
3,17,20-Trihydroxy-pregn-5-en-11-one	↑	1.407051974	0.607609343	↓	1.13634188	0.616108123	–	–	–
Methyl-ornithine	↑	1.396713015	4.289348653	–	–	–	↑	1.851698474	6.783084991
Gamma-tocopherol	↑	1.31214648	0.422588606	–	–	–	–	–	–
Myristyl myristate	↑	1.298795201	0.630153348	–	–	–	–	–	–
N-acetyl-l-tyrosine	↑	1.292701431	5.646365213	–	–	–	↑	1.740867499	6.606428793
N-acetyl-d-glucosamine	↑	1.212232395	0.600395827	↓	1.561826848	0.390920884	–	–	–
4-Fluoroaniline	↑	1.202751368	0.712701436	↓	1.338950179	0.607876328	↓	1.220492817	0.551222842
3-Methoxybenzenepropanoic acid	↑	1.202423954	0.602918446	↓	1.312977897	0.496551564	–	–	–
Hydroxylamine	↑	1.115318389	0.69697919	–	–	–	–	–	–
2-Deoxy-d-arabino-hexose 6-phosphate	↑	1.072307335	0.703428708	–	–	–	–	–	–
Beta-alanine	↑	1.03923823	0.733352214	–	–	–	–	–	–
Dehydroabietic acid	↓	1.036573447	1.639369003	–	–	–	–	–	–
Octadecanol	↑	1.021249602	0.72319414	–	–	–	–	–	–
Methyl 3-o-acetyl-d-galactopyranoside	–	–	–	↓	3.546761395	50.79637748	↑	3.219100831	95.48366296
Cadaverine	–	–	–	↑	3.249793127	26.58160269	↑	2.9534596	46.99554823
Spermine	–	–	–	↑	2.714630338	12.73155507	↑	2.649452499	19.16906612
Lanosterol	–	–	–	↑	2.502953691	8.117551506	↑	2.419124881	15.4194307
Dl-dopa	–	–	–	↑	2.278080192	8.532116661	↑	2.293110588	14.32990795
Formononetin	–	–	–	↑	2.270046002	9.822929094	↑	1.97307898	9.381507399
Glucosaminic acid	–	–	–	↓	2.181829238	0.357216013	↓	2.37047523	0.037370048
Norleucine	–	–	–	↑	2.118520585	4.598049301	↑	2.071294272	7.770456873
Isochlorogenic acid	–	–	–	↑	1.818881432	4.358736024	–	–	–
3,6-Dimethyl-2,5-dihydroxy-pyrazine	–	–	–	↑	1.777727214	4.265676317	↑	1.574528315	4.920219604
Glutaric acid	–	–	–	↑	1.450959352	3.100227715	↑	1.562242956	3.19451363
Kynurenic acid	–	–	–	↑	1.36570299	2.120110718	↑	1.257077909	2.359946098
Coniferin	–	–	–	↑	1.345755144	1.832205622	↑	1.061584725	1.764265764
Desyrel	–	–	–	↑	1.209336869	2.256585326	↑	1.386756012	3.667487452
Serine	–	–	–	↑	1.201010068	4.656242447	↑	1.338436228	4.379419202
l-tyrosine	–	–	–	↑	1.2007011	4.470832506	↑	1.325122374	4.15573751
Cholest-4-en-3beta-ol	–	–	–	↑	1.115271504	2.067697748	↑	1.062302859	1.747385641
3-Ureidopropionate	–	–	–	↑	1.062646356	2.177947513	↑	1.176974319	2.254195005
Xanthurenic acid	–	–	–	–	–	–	↑	2.872893529	38.14015946
2-Methylpropan-2-ol	–	–	–	–	–	–	↑	2.208536317	10.904428
Linoleic acid	–	–	–	–	–	–	↑	2.115009437	7.006437266
Erucic acid	–	–	–	–	–	–	↑	2.090320933	6.855409628
Oleic acid	–	–	–	–	–	–	↑	2.088193053	6.834527523
Uridine	–	–	–	–	–	–	↑	1.936972667	8.557614511
Methanephosphonothioic acid	–	–	–	–	–	–	↑	1.890927922	9.474598911
1-Monoolein	–	–	–	–	–	–	↑	1.885301741	5.150910645
Lactitol	–	–	–	–	–	–	↑	1.880504466	9.535811475
l-phenylalanine	–	–	–	–	–	–	↑	1.807190758	8.427881632
Harmol	–	–	–	–	–	–	↑	1.712406045	4.132705594
D-myo-inositol 4-phosphate	–	–	–	–	–	–	↑	1.707871292	4.047956929
Beta-gentiobiose	–	–	–	–	–	–	↑	1.699891061	9.573992054
l-asparagine	–	–	–	–	–	–	↑	1.667700919	7.669235485
Cis-gondoic acid	–	–	–	–	–	–	↑	1.653168498	3.519587343
Mannose	–	–	–	–	–	–	↑	1.561349611	5.546406513
5,6-Dihydrouracil	–	–	–	–	–	–	↑	1.550605861	3.104286803
Arabinofuranose	–	–	–	–	–	–	↑	1.54794654	3.636328597
Glucose	–	–	–	–	–	–	↑	1.54268524	5.636682124
Phosphoenolpyruvate	–	–	–	–	–	–	↑	1.533125251	5.116154387
2,5-Dihydroxy-pyrazine	–	–	–	–	–	–	↑	1.526759461	3.48422447
l-methionine	–	–	–	–	–	–	↑	1.485532306	8.132759572
5-Phenyl-2,4-pyrimidinediamine	–	–	–	–	–	–	↑	1.43991445	2.514418711
Hydrocinnamic acid	–	–	–	–	–	–	↑	1.400430369	4.305094303
l-lysine	–	–	–	–	–	–	↑	1.370847431	11.43132343
d-ribose	–	–	–	–	–	–	↑	1.28869113	2.476327512
Pinitol	–	–	–	–	–	–	↓	1.254542727	0.436613123
Ethanolamine	–	–	–	–	–	–	↑	1.220236118	2.744046409
1-Methylinosine	–	–	–	–	–	–	↑	1.20580382	2.272349749
Tert-butylketene acetal	–	–	–	–	–	–	↑	1.1714272	2.225041289
Citrulline	–	–	–	–	–	–	↑	1.167472522	3.958115816
2-Hydroxyundecanoic acid	–	–	–	–	–	–	↑	1.129282122	3.006944596
l-valine	–	–	–	–	–	–	↑	1.120861129	2.533242113
5′-Deoxy-5′-methylthioadenosine	–	–	–	–	–	–	↑	1.105423245	2.024462065
Acebutolol	–	–	–	–	–	–	↓	1.07695027	0.528863204
l-alanine	–	–	–	–	–	–	↑	1.047352349	2.710424916

↑: Upregulated; ↓: downregulated. (V, variation; VIP, variable importance in the projection; FC, fold change; P, pulmonary infections; A, abdominal infections; U, urinary tract infections; H, healthy controls).

### Differential metabolites as candidate biomarkers distinguish sepsis from healthy controls

**Figure 4:**
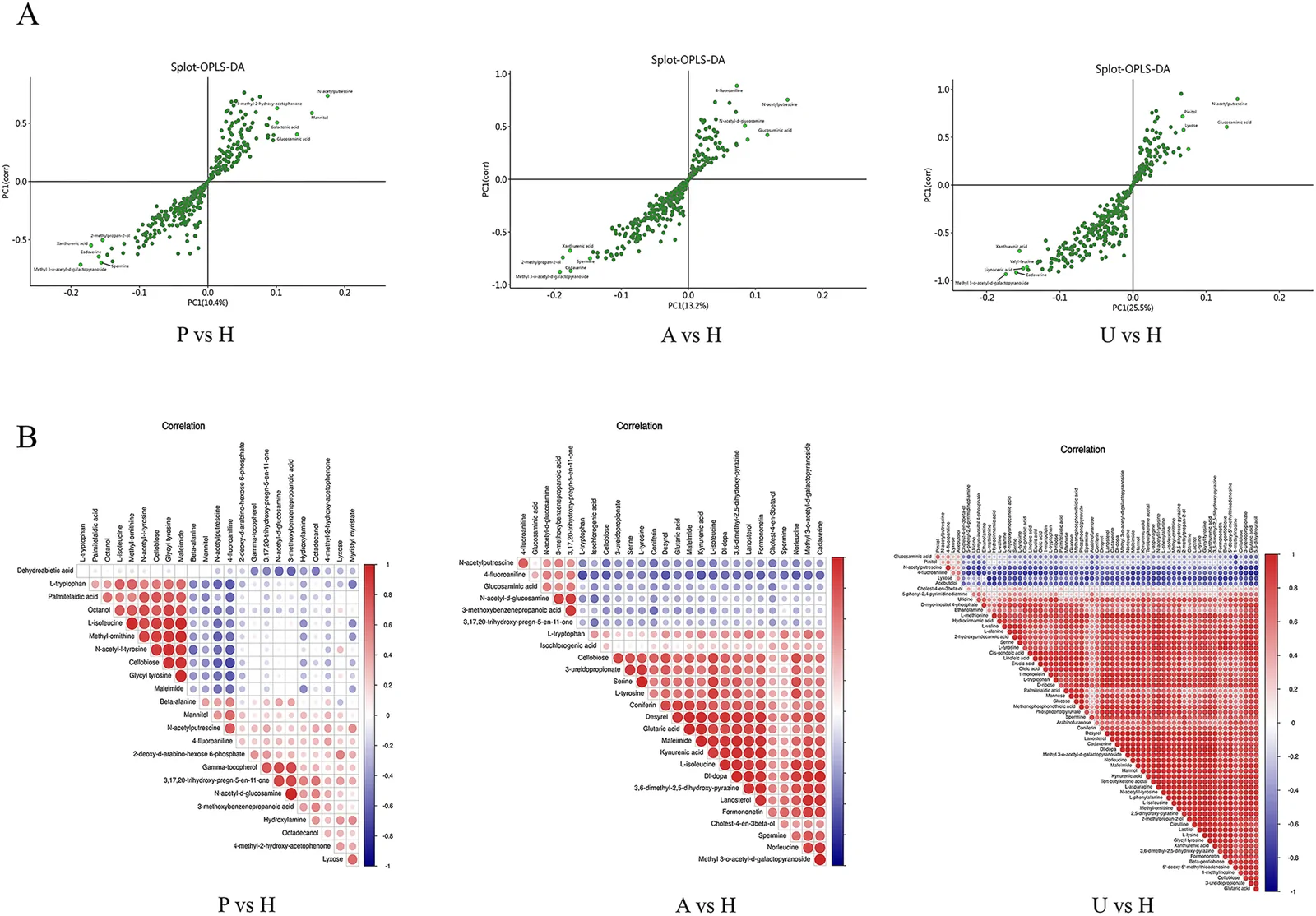
The S-plot was used to screen the potential biomarkers distinguishing between sepsis patients and healthy controls and the correlation between differential metabolites. A: The S-plot was used to select the potential biomarkers distinguishing between sepsis patients and healthy controls. B: Correlation analysis was used to measure the degree of correlation between differential metabolites. The red dots indicated positive correlation, and the blue dots indicated negative correlation. P: pulmonary infections; A: abdominal infections; U: urinary tract infections; H: healthy controls.

To identify key metabolites distinguishing sepsis patients from healthy controls, we leveraged the S-plot from our OPLS-DA models. In this plot, the abscissa represents the contribution of metabolites (covariance), while the ordinate indicates its confidence (correlation). Metabolites with greater discriminatory power are located farther into the first or third quadrant, forming an “S” shape. Analysis of the S-plots across all infection types consistently identified two metabolites, methyl 3-o-acetyl-d-galactopyranoside and N-acetylputrescine, as the most significant, positioning them as candidate biomarkers for sepsis (Figure 4A). A significant negative correlation was subsequently confirmed between these two metabolites using Pearson correlation analysis (Figure 4B). We also analysis the diferential metabolites among subgroups, the results were shownin Figure S1.

### Metabolic pathway enrichment in sepsis subgroups

**Figure 5:**
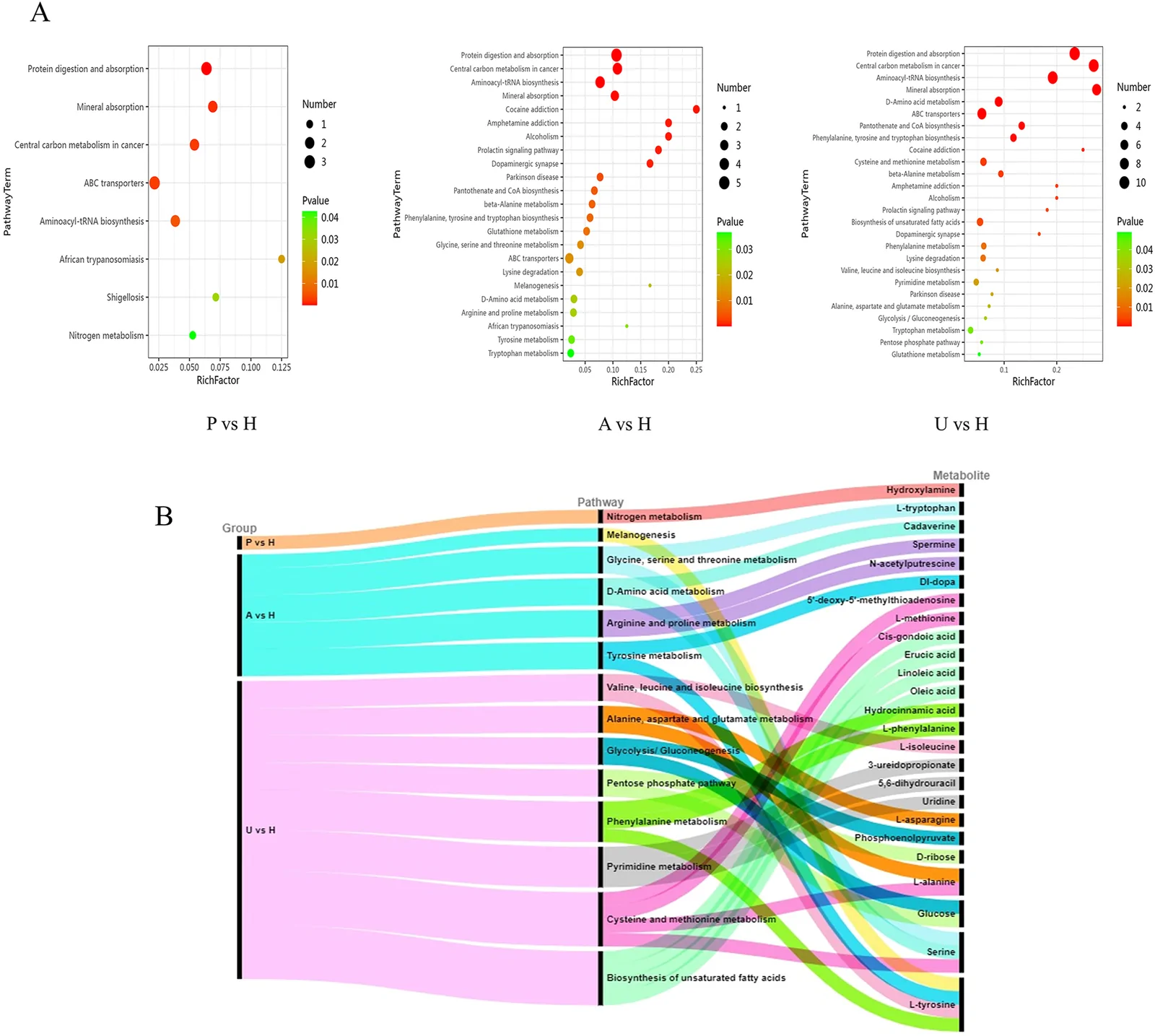
The metabolic pathways were enriched by KEGG. A: The bubble diagram was analyzed using KEGG metabolism pathway enrichment. The p value is the significance of enrichment of this metabolic pathway. The ordinate is the name of the metabolic pathway; the abscissa is the rich factor (rich factor=the number of significantly different metabolites/the total number of metabolites in this pathway). The larger the rich factor, the more metabolites enriched in the pathway. The color from green to red indicates that p-value decreases in turn. B: Sankey plot showing the correlations among pathways and differentially expressed metabolites. P: pulmonary infections; A: abdominal infections; U: urinary tract infections; H: healthy controls.

To elucidate the metabolic pathways altered in different types of sepsis, we performed KEGG pathway enrichment analysis on the identified differential metabolites (Figure 5, Figure S2). The results revealed distinct pathway profiles across the infection sites.

Pulmonary infection: Compared to healthy controls, eight pathways were enriched. Notably, nitrogen metabolism was uniquely enriched in this subgroup.

Abdominal infection: 23 pathways were enriched. Four pathways, glycine, serine and threonine metabolism; melanogenesis; arginine and proline metabolism; and tyrosine metabolism, were unique to abdominal infection and primarily involved in amino acid metabolism. The candidate biomarker N-acetylputrescine was enriched in the arginine and proline metabolism pathway, underscoring its potential diagnostic relevance for this subtype.

UTI: 26 pathways were enriched. UTI patients exhibited five unique pathways associated with the use of glucocorticoids and catecholamines, (e.g. cocaine and amphetamine addiction; alcoholism; prolactin signaling pathway; dopaminergic synapses) and eight distinct metabolic pathways. These included pathways for amino acid biosynthesis and metabolism (cysteine and methionine metabolism; phenylalanine metabolism; valine, leucine and isoleucine biosynthesis; alanine, aspartate and glutamate metabolism), biosynthesis of unsaturated fatty acids, pyrimidine metabolism, and key glucose metabolism pathways (glycolysis/gluconeogenesis and the pentose phosphate pathway). In summary, compared with the other two infections mentioned above, UTI was uniquely characterized by alterations in glucose and pyrimidine metabolism, in addition to changes in amino acid-related pathways. The enrichment of glucose metabolism pathways is consistent with our clinical finding of elevated blood glucose levels in UTI patients (Table 1), highlighting their critical role in this subtype sepsis.

### Differential metabolites can distinguish and serve as predictive biomarkers for different types of infection in patients with sepsis

**Figure 6:**
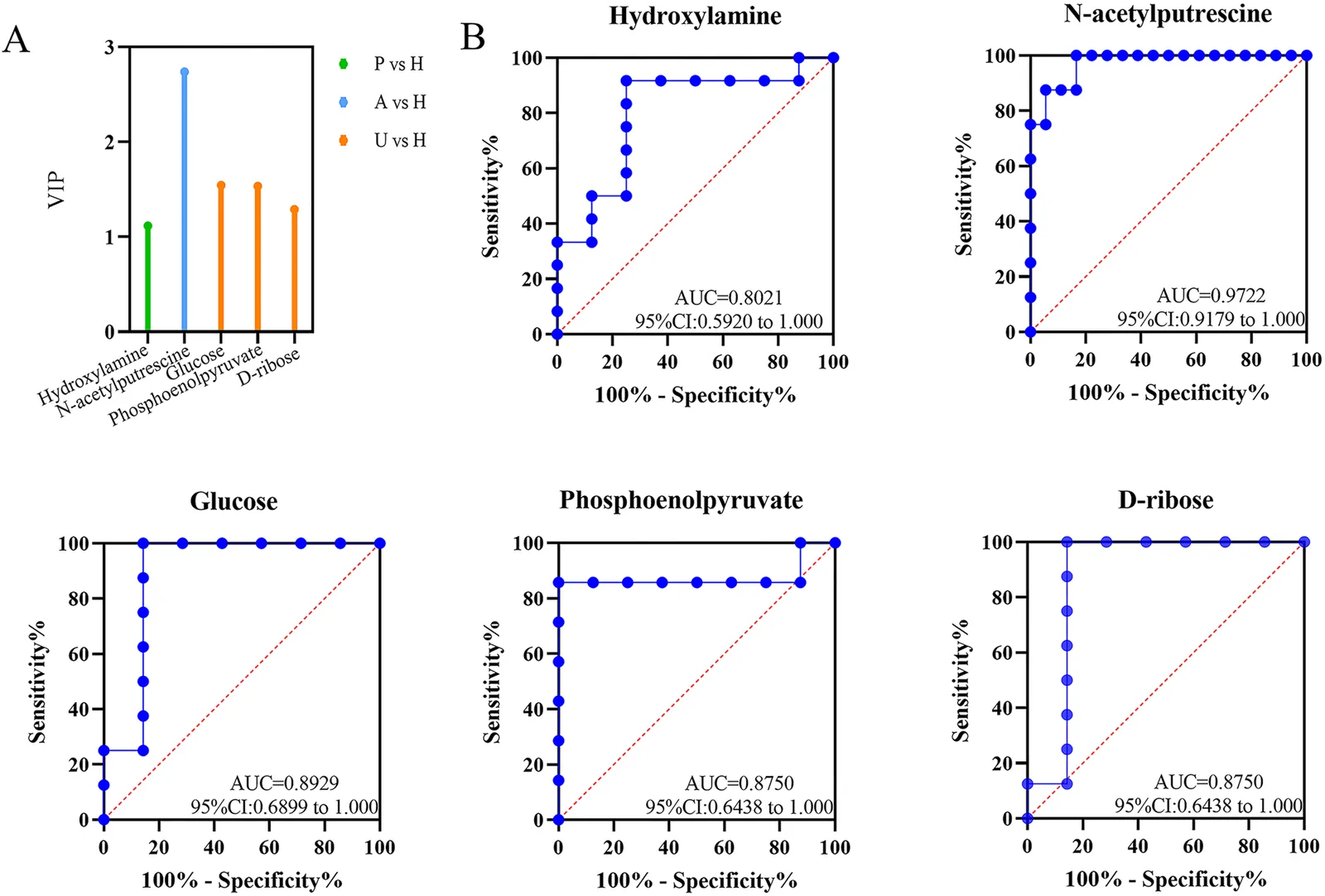
The ROC was used to analyze the potential biomarkers between patients. A: The lolipopmap of the VIP of differential metabolites between groups. B: The ROC was used to analyze the potential biomarkers for distinguishing different kinds of infection. Abbreviations: ROC, receiver operating characteristic.

ROC analysis identified simplified diagnostic biomarkers for distinguishing sepsis patients with pulmonary, abdominal, or UTI from healthy controls. The area under the curve (AUC) of each candidate metabolite was calculated. As illustrated in the lolipop map (Figure 6A), hydroxylamine best predicted pulmonary infection (AUC=0.8021, 95 % confidence interval:0.5920 to 1.000), while N-acetylputrescine was the top predictor for abdominal infection (AUC=0.9722, 95 % confidence interval:0.9179 to 1.000). For UTI, glucose (AUC=0.8929), phosphoenolpyruvate (AUC=0.875), and d-ribose (AUC=0.875) demonstrated strong diagnostic performance (Figure 6B).

### The correlation between candidate metabolites and clinical indicators

**Figure 7:**
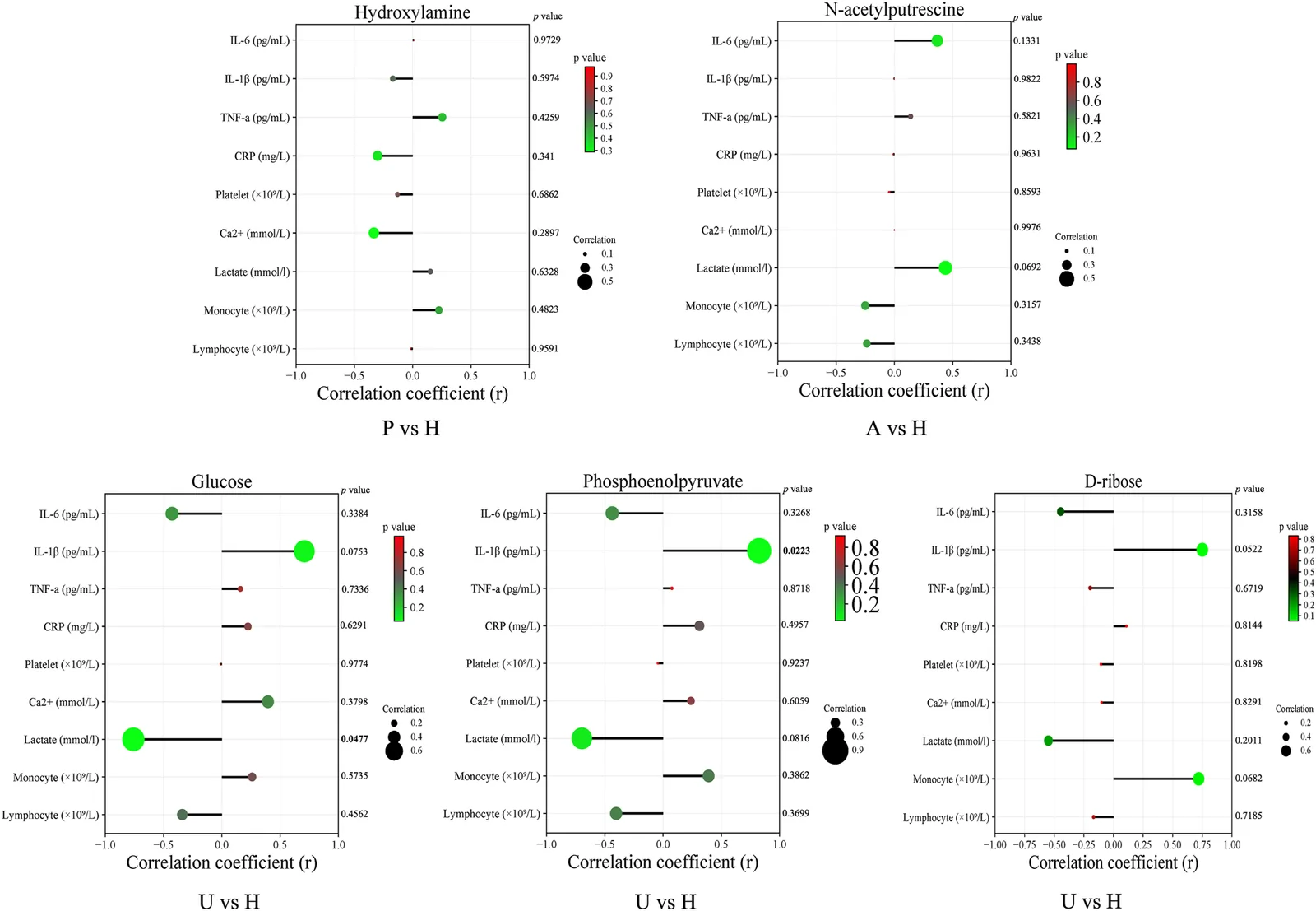
The correlation between candidate metabolites and clinical indicators. The lolipopmap demonstrated the correlation between candidate metabolites and clinical indicators. Abbreviations: IL-6: interleukin-6; IL-1β: interleukin-1β; TNF-α: tumor necrosis factor-α; CRP: c-reactive protein. P: pulmonary infections; A: abdominal infections; U: urinary tract infections; H: healthy controls.

To further assess the clinical relevance of the candidate metabolites, we analyzed their correlations with various clinical indicators. The analysis revealed that neither hydroxylamine nor N-acetylputrescine showed significant correlations with indicators including interleukin-6 (IL-6), IL-1β, tumor necrosis factor-α (TNF-α), CRP, platelet count, Ca^2+^, monocyte count, or lymphocyte count. In contrast, glucose exhibited a significant negative correlation with lactate (r=−0.7594, p=0.0477), while phosphoenolpyruvate was positively correlated with IL-1β (r=0.8251, p=0.0223) (Figure 7).

### Post hoc statistical power of the present cohort

Given the subgroup sizes, the study had 80 % power at α=0.05 to detect only large standardized differences: Cohen’s d ≥1.35 for pulmonary infection vs. controls, d≥1.25 for abdominal infection vs. controls, and d≥1.53 for UTI vs. controls. For the candidate biomarkers with the largest observed effects, the achieved power was correspondingly high-for example, N-acetylputrescine in abdominal infection (AUC=0.9722) reached >90 % power against the null AUC of 0.5. In contrast, hydroxylamine in pulmonary infection (AUC=0.8021, 95 % CI 0.5920-1.000) and the UTI discriminators glucose (AUC=0.8929) and phosphoenolpyruvate and d-ribose (AUC=0.875) were supported by an estimated power of only approximately 55–65 %, and their confidence intervals extend close to, or approach, the non-informative boundary. Metabolites with more moderate fold changes were therefore likely to be missed (type II error), and the wide confidence intervals indicate that the point estimates of accuracy are imprecise. Full details are provided in Table S2.

## Discussion

Although sepsis has been recognized for a long time, it remains a major challenge in critical care medicine due to its high heterogeneity, diverse causes, diverse clinical manifestations, rapid progression, and high mortality. Early diagnosis facilitated by biomarkers could enable timely intervention, potentially reducing mortality. While numerous biomarkers have been proposed [9], current standards like PCT and CRP alone or combined with TRAIL and IP-10 have limitations in accurately identifying infection types (bacterial or viral). There has been significant advancement in genomic and molecular methodologies that enable precise detection of bacterial RNA/DNA in body fluids [10]. During recovery from septic shock, changes in the expression of key inflammatory proteins and genes S100A8 and S100A12 were found to be inversely correlated with the expression of CD74 and HLA-DR [11]. Encouragingly, emerging approaches in sepsis research, analogous to biomarker-driven enrichment strategies used in oncology for immune or targeted therapies, are being developed based on the evaluation of biomarkers such as sTREM-1, bio-adrenomedullin, and monocyte HLA-DR expression. These biomarkers reflect specific pathophysiological mechanisms in certain sepsis patients and hold promise for personalized treatment [12], [13], [14].

Metabolomics has recently emerged as a powerful approach for identifying novel sepsis biomarkers. Our previous GC-MS-based metabolomic study identified novel diagnostic and predictive biomarkers for sepsis and SIRS [8]. Specifically, methyl 3-O-acetyl-d-galactopyranoside and N-acetylputrescine were identified as candidate biomarkers for distinguishing patients with SIRS or sepsis from healthy controls, while 2-deoxy-d-erythro-pentofuranose-5-phosphate emerged as a potential biomarker for discriminating sepsis from SIRS. Given the urgent need for novel and specific sepsis biomarkers, the present study further explored metabolic differences associated with distinct infection sources in patients with established sepsis. We demonstrated that sepsis patients with different infection sources exhibit distinct metabolomic profiles compared to healthy controls.

Specifically, our study showed, glucose, phosphoenolpyruvate, and d-ribose effectively discriminated sepsis patients with UTI from healthy controls. KEGG pathway analysis linked these metabolites to glycolysis/gluconeogenesis and the pentose phosphate pathway. This aligns with the established “Warburg effect” in sepsis in previous studies, where glycolytic flux is heightened. Glucose undergoes glycolysis to produce phosphoenolpyruvate, which further produces pyruvate and ATP, providing energy for the body [15]. Furthermore, our findings indicate an enrichment of the pentose phosphate pathway in septic patients with UTI. The pentose phosphate pathway generates reduced form of nicotinamide adenine dinucleotide phosphate (NADPH) and H+ [16], 17], which plays a critical role in biosynthesis, hydroxylation, and antioxidant defense. Additionally, it supplies essential substrates for glycolysis and cellular energy production. Consequently, elevated levels of metabolites such as glucose, phosphoenolpyruvate, and d-ribose may indicate the presence of UTI in sepsis, offering potential diagnostic clues for clinicians.

The production of N-acetylputrescine by *Escherichia coli* (*E. coli*) has been previously reported [18]. As a common member of the human gut microbiota and a frequent opportunistic pathogen, *E. coli* can translocate into the bloodstream during sepsis due to a compromised intestinal barrier. Our study identified N-acetylputrescine as the top predictor for abdominal infection. Furthermore, this metabolite was detectable in the blood of septic patients with intestinal infections, where it is likely produced by translocated *E. coli*. Therefore, N-acetylputrescine represents a reliable indicator for identifying sepsis patients with concurrent intestinal infection.

In our study, hydroxylamine was identified as the top metabolite distinguishing septic patients with pulmonary infection from healthy controls. Hydroxylamine plays a crucial role in the nitrogen cycle and metabolism of nitrogen-containing compounds in living organisms. It participates in the synthesis of NADPH as an NAD+ oxidoreductase [19]. Two molecules of hydroxylamine combined with two NAD+ can be converted into hyponitrite, along with two NADH and two H+. NADPH is the reduced form of NADP+, and serves as a common coenzyme and biological reducing agent, produced through the pentose phosphate pathway or photosynthesis. NADPH acts as an electron donor in the body, participating in the synthesis and metabolism of lipids, fatty acids, and nucleotides, and plays a key role in maintaining redox balance and resisting damage from reactive oxygen species.

Analysis of the correlation between candidate metabolites and clinical indicators revealed a negative association between glucose and lactate, which can be explained by the glycolytic breakdown of glucose into lactate. Furthermore, our results demonstrated a positive correlation between phosphoenolpyruvate and IL-1β. Since phosphoenolpyruvate is an intermediate in the glycolytic pathway, its accumulation, particularly under anaerobic glycolysis, was found to be associated with UTI in sepsis patients. Elevated levels of phosphoenolpyruvate were correlated with increased severity of UTI.

This study has several limitations. First and most importantly, the cohort was small and the post hoc analysis confirmed that it was adequately powered only for large effects (minimum detectable Cohen’s d of 1.25–1.53 across subgroups; Table S2). Two consequences follow. On the one hand, metabolites with genuine but moderate differences were probably not detected, so the reported panels are unlikely to be exhaustive. On the other hand, in small samples the magnitude of a significant effect tends to be overestimated, and the AUC values reported here, particularly for hydroxylamine in pulmonary infection and for the UTI discriminators, where the UTI subgroup comprised only seven patients and achieved power was approximately 60 %, should be regarded as upper-bound estimates with wide confidence intervals rather than as stable measures of diagnostic accuracy. In addition, the differential metabolites were screened using nominal p-values combined with VIP>1.0, without formal false-discovery-rate correction; with a panel of this size, some findings may reflect type I error. Consistent with this, although our OPLS-DA models showed high R^2^Y(cum) (0.994-0.997), the Q^2^(cum) values (0.757-0.877) and the negative Q^2^ intercepts of the permutation tests are typical of models built on small sample sets and indicate limited predictive generalizability. For these reasons we present the candidate metabolites as exploratory, hypothesis-generating findings rather than as validated diagnostic markers.

To address this directly, we have estimated the sample size required for the planned validation study. Detecting a moderate effect (d=0.8) at 80 % power and α=0.05 requires approximately 26 participants per group, and detecting d=0.5 requires approximately 64 per group; constraining the 95 % CI of an AUC of 0.80 to a half-width of about±0.10 requires roughly 45 cases and 45 controls. We therefore plan a prospective cohort of at least 40 patients for each infection subtype together with 40 non-septic critically ill controls, i.e. approximately 160 participants in total. Relative to the present study, this design would lower the minimum detectable effect size by roughly a factor of two, permit Benjamini-Hochberg control of the false-discovery rate across the metabolite panel while retaining adequate power, allow the cohort to be split into training and independent test sets for internal validation of the OPLS-DA and ROC models, and provide sufficient degrees of freedom for multivariable adjustment for age, sex, comorbidity, and glycemic status -confounders that the current subgroups, which differ markedly in sex distribution and in blood glucose, cannot be adjusted for. Second, Given the high heterogeneity of sepsis, integrating metabolomic and clinical data through weighted gene co-expression network analysis (WGCNA) would help relate systemic metabolic disturbances to specific clinical trajectories. As WGCNA requires a larger sample size for robust module detection, we will apply this framework in an expanded cohort to provide further mechanistic and precision-diagnostic insights. Forth, External validation in independent cohorts was not available in this study; confirming these candidate biomarkers in independent external datasets is important in next step. A further limitation concerns the quantification strategy. Because a single, non-isotope-labeled internal standard 2-chloro-l-phenylalanine was used for all analytes, the ionization detector response, which is strongly dependent on the chemical and physicochemical properties of each compound, was not individually corrected. As a result, relative peak-area ratios may not faithfully reflect the true concentration relationships among different metabolites, and the reported fold changes should be interpreted as relative differences of a given metabolite between groups rather than absolute concentration differences. Future validation should employ targeted, absolutely quantified assays using authentic standards and compound-specific stable-isotope-labeled internal standards to confirm the candidate biomarkers.

## Conclusions

Our GC-MS-based metabolomic study identified potential biomarkers for sepsis across various infection types. These candidate biomarkers may help characterize and classify infection-specific metabolic profiles in patients already diagnosed with sepsis. They represent exploratory findings rather than validated diagnostic or treatment-response markers; their clinical utility requires prospective validation in larger and independent cohorts.

## Supplementary Material

Supplementary Material
