# Supplementary material for: GC-MS metabolomics reveals potential biomarkers for infection-specific subtypes in sepsis
Source: Open Med (Wars). 2026 Sep 24;21(1):20261539. doi: 10.1515/med-2026-1539 (PMC13603071; doi:10.1515/med-2026-1539)
Supplement: Supplementary file 1 [file j_med-2026-1539_suppl_001.docx]

**Supplementary Materials**

**Figure S1**. Differential metabolites were compared between subgroups by the histogram.

**Figure S2**. The metabolic pathways were enriched by KEGG between subgroups. No metabolic pathways were enriched between pulmonary infection group and abdominal infection group.

**Table S1.** MS parameters for each metabolite.

| **Alignment ID** | **Average Rt(min)** | **Quant mass** | **Metabolite name** | **Total score** | **HMDB** |
| --- | --- | --- | --- | --- | --- |
| 0 | 6.34 | 121.0975 | Boric acid | 93.7 |  |
| 1 | 6.399 | 147.08 | Aconitic acid | 79.9 | HMDB0000072 |
| 2 | 6.455 | 156.1251 | Piperidone | 87.5 | HMDB0011749 |
| 3 | 6.494 | 147.0712 | 4-hydroxybutyric acid | 84.3 | HMDB0000710 |
| 6 | 6.594 | 147.05 | Homovanillic acid | 65.5 | HMDB0000118 |
| 17 | 6.809 | 174.1278 | 1-propylamine | 97.1 | HMDB0034006 |
| 20 | 6.868 | 136.0672 | 2-picolinic acid | 78.7 | HMDB0002243 |
| 22 | 6.901 | 147.0726 | Sulfuric acid | 85.7 | HMDB0001448 |
| 31 | 7.132 | 221.0695 | 2-mercapto-4,6-dimethylnicotinonitrile | 78.9 |  |
| 36 | 7.295 | 318.0978 | Pyruvic acid | 76.4 | HMDB0000243 |
| 37 | 7.311 | 166.075 | Phenol | 78.3 | HMDB0000228 |
| 39 | 7.365 | 174.1136 | Diethylcarbamic acid | 97.5 |  |
| 41 | 7.428 | 214.1283 | 5-hydroxynorvaline | 72.1 |  |
| 42 | 7.481 | 147.08 | Carbamic acid | 86.9 | HMDB0003551 |
| 45 | 7.488 | 117.1167 | L-lactic acid | 99.5 | HMDB0000190 |
| 50 | 7.639 | 210.07 | 2,3-dihydro-8-methoxyfuro(2,3-b)quinoline | 67.4 |  |
| 55 | 7.723 | 147.0725 | Glycolic acid | 97 | HMDB0000115 |
| 56 | 7.759 | 169.1288 | Thymidine | 70.1 | HMDB0000273 |
| 57 | 7.808 | 200.1252 | Kynurenic acid | 73.3 | HMDB0000715 |
| 60 | 7.864 | 318.1 | Hexitol | 75.2 | HMDB0000107 |
| 62 | 7.873 | 146.0775 | L-valine | 98.9 | HMDB0000883 |
| 63 | 7.874 | 147.0763 | Citraconic acid | 78.2 | HMDB0000634 |
| 64 | 7.958 | 154.0419 | Maleimide | 87.7 |  |
| 65 | 7.99 | 174.1362 | 1-butylamine | 88.8 | HMDB0031321 |
| 68 | 8.042 | 240.09 | 2-ketoisovaleric acid | 72 | HMDB0000019 |
| 69 | 8.062 | 113.0995 | 2,4-diaminobutyric acid | 72.8 | HMDB0002362 |
| 70 | 8.082 | 85.1 | Hexadecane | 76.9 | HMDB0033792 |
| 74 | 8.198 | 116.1097 | L-alanine | 95.5 | HMDB0000161 |
| 81 | 8.397 | 133.0596 | Hydroxylamine | 98 | HMDB0003338 |
| 83 | 8.448 | 126.1871 | Adrenaline | 82.7 | HMDB0014344 |
| 87 | 8.541 | 147.07 | Phthalic acid | 78.4 | HMDB0002107 |
| 88 | 8.542 | 258.1402 | 2,5-piperazinedione | 77.9 |  |
| 89 | 8.599 | 168.0572 | 4-fluoroaniline | 73.5 |  |
| 90 | 8.626 | 245.1235 | Tert-butylketene acetal | 77.3 |  |
| 92 | 8.671 | 130.1167 | L-alanine-alanine | 83.5 |  |
| 93 | 8.726 | 152.0604 | 3-pyridinol | 91.2 |  |
| 95 | 8.748 | 191.1075 | 3-hydroxybutyric acid | 84.7 | HMDB0000357 |
| 97 | 8.837 | 155.063 | Benzylalcohol | 78 | HMDB0003119 |
| 113 | 9.184 | 191.115 | (r)-3-hydroxybutyric acid | 98.9 | HMDB0000011 |
| 114 | 9.225 | 152.0584 | Propane-1,3-diol | 77.6 |  |
| 118 | 9.355 | 103.03 | 4-methyl-5-thiazoleethanol | 79.1 | HMDB0032985 |
| 119 | 9.361 | 280.96 | 4,4-dimethyl-1,3-diphenyl-1-hydroxy-1-pentene | 76.8 |  |
| 120 | 9.389 | 130.1096 | N-methylalanine | 88.7 | HMDB0094692 |
| 121 | 9.391 | 130.0914 | Homoserine | 77.2 | HMDB0000719 |
| 122 | 9.399 | 86.12 | 2-methylpropan-2-ol | 86.2 | HMDB0031456 |
| 123 | 9.465 | 187.1443 | N-acetyl-d-tryptophan | 72.5 | HMDB0013713 |
| 126 | 9.531 | 89.055 | Phosphoenolpyruvate | 85.1 | HMDB0000263 |
| 127 | 9.537 | 241.085 | Methanephosphonothioic acid | 85.7 |  |
| 130 | 9.633 | 187.06 | Octanol | 72.5 | HMDB0001183 |
| 136 | 9.737 | 249.1454 | Epicatechin | 74.7 | HMDB0001871 |
| 137 | 9.738 | 249.1426 | Ferulic acid | 71.8 | HMDB0000954 |
| 141 | 9.893 | 206.0786 | Hippuric acid | 71.6 | HMDB0000714 |
| 147 | 10.028 | 219.083 | 3,4-dihydroxycinnamic acid | 77.4 | HMDB0001964 |
| 152 | 10.109 | 174.09 | Mannitol | 74.7 | HMDB0000765 |
| 153 | 10.143 | 86.09333 | 3-ureidopropionate | 73.9 | HMDB0000026 |
| 161 | 10.226 | 172.17 | Glycocyamine | 71.2 | HMDB0000128 |
| 164 | 10.301 | 112.116 | Dehydroascorbic acid | 82.2 | HMDB0001264 |
| 170 | 10.413 | 266.1581 | Hypoxanthine | 75 | HMDB0000157 |
| 173 | 10.487 | 147.08 | Ascorbic acid | 83.1 | HMDB0000044 |
| 176 | 10.555 | 187.05 | Ricinoleic acid | 68.9 | HMDB0034297 |
| 179 | 10.628 | 179.0637 | Benzoic acid | 90 | HMDB0001870 |
| 185 | 10.755 | 231.162 | 3,6-anhydro-d-galactose | 86.2 |  |
| 186 | 10.795 | 102.0849 | Dodecanoic acid, isopropanol ester | 77.1 | HMDB0059820 |
| 187 | 10.814 | 189.11 | Urea | 97.3 | HMDB0000294 |
| 188 | 10.815 | 88.05231 | Xylonolactone | 73.9 | HMDB0011676 |
| 191 | 10.88 | 85.11352 | Dihydroxyacetone | 90.8 | HMDB0001882 |
| 194 | 10.906 | 117.057 | 2-deoxyerythritol | 85 |  |
| 195 | 10.921 | 201.1511 | Octanoate radical | 96.2 | HMDB0062511 |
| 196 | 10.966 | 192.1036 | 3-(4-hydroxyphenyl)propionic acid | 78 | HMDB0002199 |
| 197 | 10.974 | 174 | Tranexamic acid | 89.6 | HMDB0014447 |
| 198 | 11.022 | 174.12 | N-acetylornithine | 93.2 | HMDB0003357 |
| 199 | 11.041 | 175.23 | Ethylsuccinate | 75.8 | HMDB0061930 |
| 200 | 11.062 | 180.0531 | Nicotinic acid | 70.4 | HMDB0001488 |
| 202 | 11.082 | 174.1271 | Ethanolamine | 98.1 | HMDB0000149 |
| 207 | 11.283 | 299.1883 | (4-hydroxy-3-methoxyphenyl)ethanol | 89.6 | HMDB0038925 |
| 208 | 11.285 | 299.1872 | Bisphenol a monomethyl ether | 89.5 |  |
| 209 | 11.296 | 300.1703 | Ciliatine | 94.7 | HMDB0011747 |
| 211 | 11.301 | 205.1257 | Glycerol | 90 | HMDB0000131 |
| 214 | 11.418 | 158.1215 | Trans-4-hydroxyproline | 81.8 | HMDB0000725 |
| 216 | 11.457 | 131.0922 | 2-hydroxyvaleric acid | 77.8 | HMDB0001863 |
| 219 | 11.519 | 280.164 | 2'-deoxyguanosine | 75.9 | HMDB0000085 |
| 223 | 11.614 | 158.155 | L-isoleucine | 95.7 | HMDB0000172 |
| 225 | 11.639 | 142.1223 | L-proline | 95.2 | HMDB0000162 |
| 226 | 11.669 | 174.14 | 5-aminovaleric acid | 85.5 | HMDB0003355 |
| 228 | 11.732 | 133 | L-cystine | 86.8 | HMDB0000192 |
| 229 | 11.738 | 176.02 | Pantothenic acid | 85.5 | HMDB0000210 |
| 232 | 11.84 | 174.19 | 3-aminoisobutanoic acid | 90.2 | HMDB0003911 |
| 233 | 11.845 | 174.1351 | Glycine | 99.4 | HMDB0000123 |
| 234 | 11.872 | 291.1152 | 4-aminobutyric acid | 82.7 | HMDB0000112 |
| 237 | 11.966 | 147.08 | Succinic acid | 89.3 | HMDB0000254 |
| 238 | 11.976 | 239.1695 | Pyrrole-2-carboxylic acid | 80.3 | HMDB0004230 |
| 241 | 12.027 | 100.1725 | Uric acid | 71.7 | HMDB0000289 |
| 246 | 12.128 | 116.07 | Sarcosine | 83.2 | HMDB0000271 |
| 252 | 12.218 | 213.156 | Glutathione | 75.4 | HMDB0000125 |
| 256 | 12.291 | 229.1567 | 1-methylhydantoin | 78.6 | HMDB0003646 |
| 257 | 12.302 | 215.125 | 2-imidazolidinone | 85 |  |
| 260 | 12.386 | 281.0753 | N-cyclohexylsulfamic acid | 79.8 | HMDB0031340 |
| 261 | 12.401 | 189.0845 | Glyceric acid | 95.1 | HMDB0000139 |
| 265 | 12.466 | 241.1144 | Uracil | 99.8 | HMDB0000300 |
| 267 | 12.524 | 239.1126 | 1,2,4-benzenetriol | 86.3 | HMDB0124831 |
| 270 | 12.556 | 245.0963 | Fumaric acid | 95.4 | HMDB0000134 |
| 272 | 12.587 | 466.1385 | 3-(3-hydroxyphenyl)-3-hydroxypropionic acid | 73.5 | HMDB0002643 |
| 275 | 12.652 | 172.1237 | N-methyl-l-leucine | 78.2 |  |
| 279 | 12.743 | 298.1225 | 3-hydroxy-3-(4'-hydroxy-3'-methoxyphenyl)propionic acid | 76.5 |  |
| 280 | 12.758 | 215.1716 | Pelargonic acid | 95.7 | HMDB0000847 |
| 282 | 12.833 | 204.08 | Shikimic acid | 83.6 | HMDB0003070 |
| 283 | 12.837 | 241.1105 | 2,5-dihydroxy-pyrazine | 89.3 |  |
| 284 | 12.838 | 207.0546 | Resveratrol | 76 | HMDB0003747 |
| 285 | 12.879 | 177.078 | Sinapinic acid | 70.4 | HMDB0032616 |
| 290 | 12.987 | 204.1527 | Serine | 96.5 | HMDB0000187 |
| 291 | 12.988 | 218.16 | L-tyrosine | 85.6 | HMDB0000158 |
| 292 | 13.018 | 204.15 | 4-(5-methyl-2-furanyl)-2-butanone | 81.8 |  |
| 296 | 13.154 | 147.06 | Erythrono-1,4-lactone | 87.3 | HMDB0000349 |
| 298 | 13.298 | 240.1128 | 1,3-dihydroxypyridine | 89.2 |  |
| 299 | 13.314 | 269.12 | 3,6-dimethyl-2,5-dihydroxy-pyrazine | 79.9 |  |
| 302 | 13.381 | 257.146 | L-cysteine-glycine | 74.6 |  |
| 305 | 13.461 | 253.1471 | Cholic acid | 70.4 | HMDB0000619 |
| 307 | 13.547 | 218.1654 | L-threonine | 99.6 | HMDB0000167 |
| 308 | 13.636 | 291.18 | Glucosaminic acid | 71.5 |  |
| 309 | 13.64 | 184.0444 | O-phosphoserine | 73.1 | HMDB0000272 |
| 310 | 13.658 | 160.0695 | Hydroquinone | 82.8 | HMDB0002434 |
| 311 | 13.663 | 221.19 | Acebutolol | 75.2 | HMDB0015324 |
| 313 | 13.731 | 243.1203 | Glutaric acid | 82.5 | HMDB0000661 |
| 314 | 13.738 | 243.129 | 5,6-dihydrouracil | 81.9 | HMDB0000076 |
| 315 | 13.746 | 255.12 | Thymine | 84.1 | HMDB0000262 |
| 317 | 13.787 | 104.0655 | Hydrocinnamic acid | 80.2 | HMDB0000764 |
| 323 | 13.979 | 270.14 | Harmol | 75.9 | HMDB0034217 |
| 328 | 14.114 | 174.1257 | Putrescine | 91.6 | HMDB0001414 |
| 329 | 14.139 | 160.0967 | N-carbamoylaspartate | 93.8 | HMDB0000828 |
| 331 | 14.201 | 155.9 | Pipecolic acid | 86.5 | HMDB0000070 |
| 333 | 14.248 | 174.114 | Beta-alanine | 91.7 | HMDB0000056 |
| 340 | 14.487 | 156.0724 | L-glutamine | 80.1 | HMDB0000641 |
| 341 | 14.497 | 86.06 | Spermidine | 83.5 | HMDB0001257 |
| 343 | 14.529 | 158.14 | Norleucine | 84.1 | HMDB0001645 |
| 348 | 14.673 | 326.9909 | Cerotinic acid | 75.2 | HMDB0002356 |
| 350 | 14.727 | 229.1906 | Capric acid | 99.5 | HMDB0000511 |
| 351 | 14.796 | 252.0817 | Arachidic acid | 80.5 | HMDB0002212 |
| 353 | 14.836 | 174.1257 | N-acetylputrescine | 91.1 | HMDB0002064 |
| 358 | 14.979 | 350.1768 | Phosphenodiimidic amide | 79.6 |  |
| 359 | 15.02 | 174.12 | Noradrenaline | 83.7 | HMDB0000216 |
| 362 | 15.06 | 205.125 | Erythrose | 92.1 | HMDB0002649 |
| 363 | 15.091 | 234.16 | Scopoletin | 74.7 | HMDB0034344 |
| 364 | 15.139 | 410.1271 | Dimethyluric acid | 65.9 | HMDB0001857 |
| 366 | 15.177 | 211.0278 | L-leucine | 77.6 | HMDB0000687 |
| 367 | 15.185 | 204.1486 | Phytosphingosine | 85.8 | HMDB0004610 |
| 370 | 15.247 | 147.07 | Aminomalonate | 88.4 | HMDB0001147 |
| 371 | 15.293 | 85.11 | Beta-glutamic acid | 79.6 |  |
| 375 | 15.408 | 128.0762 | Homocystine | 83.1 | HMDB0000575 |
| 377 | 15.472 | 85.11472 | Tetracosane | 92.6 | HMDB0034282 |
| 384 | 15.632 | 234.12 | N-acetyl-d-mannosamine | 79.7 | HMDB0001129 |
| 385 | 15.639 | 157.1 | N-acetylglutamate | 86.6 | HMDB0001138 |
| 387 | 15.688 | 147.0775 | Malic acid | 96.6 | HMDB0000744 |
| 389 | 15.767 | 174.1249 | Serotonin | 85.5 | HMDB0000259 |
| 391 | 15.8 | 213.93 | Diclofenac | 77.6 | HMDB0014724 |
| 392 | 15.842 | 218.1611 | Dl-dopa | 82.2 | HMDB0000609 |
| 393 | 15.865 | 142.96 | Phytol | 87.6 | HMDB0002019 |
| 394 | 15.928 | 174.1292 | 5-methoxytryptamine | 88.9 | HMDB0004095 |
| 395 | 15.929 | 129.11 | Acrylic acid | 83.2 | HMDB0031647 |
| 397 | 16.03 | 147.07 | D-tagatose | 72.8 | HMDB0003418 |
| 402 | 16.195 | 176.1064 | L-methionine | 95.9 | HMDB0000696 |
| 403 | 16.244 | 156.1014 | Pyroglutamic acid | 94.1 | HMDB0000267 |
| 404 | 16.257 | 147.08 | Dihydroxymalonic acid | 78.9 |  |
| 407 | 16.282 | 306.2035 | Tromethamine | 99.1 | HMDB0240288 |
| 408 | 16.33 | 232.148 | L-aspartic acid | 98.2 | HMDB0000191 |
| 409 | 16.385 | 374.1377 | Glycyl proline | 72 | HMDB0000721 |
| 410 | 16.398 | 158.07 | Isoxanthopterin | 65.4 | HMDB0000704 |
| 417 | 16.584 | 299.0787 | Diglycerol | 76.1 |  |
| 419 | 16.637 | 111.1043 | 2,6-bis(tert-butyl)phenol | 68.9 |  |
| 421 | 16.65 | 185.1233 | 2-piperidinobenzonitrile | 74.7 |  |
| 422 | 16.703 | 175.1297 | 5'-deoxy-5'-methylthioadenosine | 73.4 | HMDB0001173 |
| 423 | 16.751 | 383.1857 | Daidzein | 67.6 | HMDB0003312 |
| 426 | 16.928 | 192.0731 | L-kynurenine | 76.7 | HMDB0000684 |
| 428 | 16.976 | 447.1573 | 3,17,20-trihydroxy-pregn-5-en-11-one | 74.8 |  |
| 429 | 16.992 | 115.09 | Creatinine | 87.2 | HMDB0000562 |
| 430 | 17.014 | 292.1589 | Ribonic acid | 87.3 | HMDB0000867 |
| 431 | 17.022 | 174.1138 | Tyramine | 87.9 | HMDB0000306 |
| 432 | 17.026 | 220.1307 | L-cysteine | 99.7 | HMDB0000574 |
| 433 | 17.059 | 243.2275 | Dodecanol | 89.9 | HMDB0011626 |
| 435 | 17.108 | 268.11 | 7-hydroxynicotinic acid | 70.3 |  |
| 436 | 17.124 | 286.0928 | Allantoic acid | 75.8 | HMDB0001209 |
| 437 | 17.161 | 283.11 | Biphenyl | 70.3 | HMDB0034437 |
| 446 | 17.392 | 147.0725 | Erythronic acid | 96.6 | HMDB0000613 |
| 447 | 17.397 | 292.151 | Galactonic acid | 84.5 | HMDB0000565 |
| 450 | 17.488 | 247.1417 | L-2-hydroxyglutaric acid | 90 | HMDB0000694 |
| 452 | 17.509 | 186.1067 | Nicotianamine | 84.7 |  |
| 455 | 17.641 | 218.1288 | 1,5-anhydroglucitol | 84.3 | HMDB0002712 |
| 457 | 17.726 | 217.116 | Methyl-alpha-lyxofuranoside | 94.5 |  |
| 458 | 17.788 | 159.0979 | L-asparagine | 90.1 | HMDB0000168 |
| 462 | 17.873 | 188.146 | Allantoin | 87.8 | HMDB0000462 |
| 466 | 17.997 | 342.1725 | 1,2,3-trihydroxybenzene | 87.5 | HMDB0013674 |
| 468 | 18.095 | 369.08 | Catechin | 75.9 | HMDB0002780 |
| 469 | 18.143 | 147.05 | Cellobiose | 76.6 | HMDB0000055 |
| 475 | 18.416 | 246.1659 | L-glutamic acid | 99 | HMDB0000148 |
| 477 | 18.44 | 218.1331 | L-phenylalanine | 97.7 | HMDB0000159 |
| 478 | 18.491 | 147.08 | Isocitric acid | 81.4 | HMDB0000193 |
| 484 | 18.688 | 217.1219 | Coniferin | 77.3 | HMDB0013682 |
| 485 | 18.689 | 229.14 | 2-hydroxyundecanoic acid | 82.6 |  |
| 487 | 18.813 | 117.0456 | Dodecanoic acid | 95.9 | HMDB0000638 |
| 490 | 18.957 | 217.1375 | Arabinofuranose | 93.4 | HMDB0012325 |
| 493 | 19.024 | 221.11 | Alpha tocopherol | 72.2 | HMDB0001893 |
| 496 | 19.071 | 292.1588 | Tartaric acid | 86.3 | HMDB0000956 |
| 497 | 19.118 | 86.10143 | Valyl-leucine | 74.4 | HMDB0029131 |
| 501 | 19.178 | 188.0988 | Inosine-5'-monophosphate | 74.4 | HMDB0000175 |
| 504 | 19.286 | 191.1021 | Galactinol | 82.6 | HMDB0005826 |
| 508 | 19.404 | 191.1029 | 1,2,4,5-cyclohexanetetrol | 86.1 |  |
| 509 | 19.418 | 255.1864 | Chlorogenic acid | 71.1 | HMDB0003164 |
| 512 | 19.549 | 191.1041 | D-arabinose | 97.5 | HMDB0029942 |
| 513 | 19.578 | 279.1944 | 2,5-di-tert-butyl-4-(hydroxy)phenol | 85.3 |  |
| 515 | 19.651 | 217.1289 | Lyxose | 74.5 | HMDB0003402 |
| 520 | 19.848 | 217.1308 | Levoglucosan | 90.2 | HMDB0000640 |
| 522 | 19.883 | 242.06 | (2,3-di-tert-butyl-cyclopropenylidone)phosphorane | 81 |  |
| 524 | 19.99 | 154.0583 | L-histidine | 72.4 | HMDB0000177 |
| 525 | 19.996 | 147.078 | D-xylulose | 86.9 | HMDB0001644 |
| 527 | 19.998 | 103.0664 | D-ribose | 94.6 | HMDB0000283 |
| 530 | 20.147 | 211.16 | 3-ethyl-3,5,5-trimethyl-1-hydroxy-cyclohexene | 84.1 |  |
| 532 | 20.198 | 124.97 | Myristyl myristate | 76.6 |  |
| 533 | 20.209 | 210.2236 | Cholesterone | 60.4 | HMDB0000921 |
| 534 | 20.248 | 211.1 | 4-methyl-2-hydroxy-acetophenone | 88.3 |  |
| 542 | 20.494 | 109.21 | Caffeine | 71.9 | HMDB0001847 |
| 545 | 20.583 | 147.1 | Dihydrocholesterol | 72.9 | HMDB0001569 |
| 546 | 20.602 | 217.1333 | D-xylitol | 95.1 | HMDB0002917 |
| 550 | 20.716 | 299.095 | D-glyceraldehyde 3-phosphate | 92.8 | HMDB0001112 |
| 552 | 20.79 | 184.1275 | Citrulline | 72.8 | HMDB0000904 |
| 553 | 20.839 | 85.10786 | Alanyl-threonine | 91.4 | HMDB0028697 |
| 556 | 20.915 | 243.0904 | Beta-glycerophosphoric acid | 90.4 | HMDB0002520 |
| 557 | 20.921 | 232.116 | Uridine 5'-monophosphate | 79.3 | HMDB0000288 |
| 558 | 20.957 | 147.04 | Saccharopine | 76.9 | HMDB0000279 |
| 559 | 20.969 | 319.205 | Ribitol | 81 | HMDB0000508 |
| 562 | 21.041 | 174.1243 | Ornithine | 98.9 | HMDB0000214 |
| 570 | 21.314 | 154.0493 | Isomaltose | 73.9 | HMDB0002923 |
| 571 | 21.324 | 226.11 | N-{2-hydroxyethyl}tetradecan-1-amine | 84.9 |  |
| 573 | 21.374 | 217.09 | 5-methyluridine | 66.2 | HMDB0000884 |
| 576 | 21.47 | 315.13 | Dihydroxyacetone phosphate | 95.1 | HMDB0001473 |
| 579 | 21.644 | 299.0988 | Glycerol 3-phosphate | 97.6 | HMDB0000126 |
| 582 | 21.712 | 217.1052 | Glucose-1-phosphate | 90 | HMDB0000645 |
| 584 | 21.755 | 295.0988 | Terephthalic acid | 81.7 | HMDB0002428 |
| 587 | 21.808 | 292.165 | 2-keto-l-gluconate | 83.6 | HMDB0011732 |
| 589 | 21.82 | 315.1 | 5-phenyl-2,4-pyrimidinediamine | 80.8 |  |
| 591 | 21.901 | 299.1 | O-phosphoethanolamine | 98.4 | HMDB0000224 |
| 593 | 22.035 | 86.09545 | Spermine | 70.7 | HMDB0001256 |
| 596 | 22.139 | 292.1217 | Digalacturonic acid | 79.6 | HMDB0039721 |
| 602 | 22.435 | 174.1243 | Glycyl-glycine | 98.5 | HMDB0011733 |
| 605 | 22.574 | 299.1015 | 3-phosphoglyceric acid | 97.1 | HMDB0000807 |
| 612 | 22.746 | 273.124 | Citric acid | 93.7 | HMDB0000094 |
| 613 | 22.757 | 217.1362 | D-fructose | 93.4 | HMDB0000660 |
| 614 | 22.812 | 117.048 | Myristic acid | 99.1 | HMDB0000806 |
| 617 | 22.923 | 218.1433 | Udp-n-acetylglucosamine | 87.4 | HMDB0000290 |
| 619 | 23.045 | 174.1364 | Methyl-ornithine | 86.4 |  |
| 624 | 23.116 | 161.09 | Beta-gentiobiose | 63 |  |
| 627 | 23.189 | 131.0843 | Oleamide | 77.9 | HMDB0002117 |
| 630 | 23.291 | 132.0877 | Methyl 3-o-acetyl-d-galactopyranoside | 78.8 |  |
| 635 | 23.424 | 217.1378 | Xylofuranose | 85.6 |  |
| 636 | 23.461 | 290.14 | N-acetyl-5-hydroxytryptamine | 63.7 | HMDB0001238 |
| 637 | 23.505 | 157.065 | Sophorose | 85.5 |  |
| 639 | 23.669 | 179.103 | N-acetyl-l-tyrosine | 85.4 | HMDB0000866 |
| 641 | 23.717 | 345.21 | Quinic acid | 82.4 | HMDB0003072 |
| 643 | 23.748 | 217.1258 | Pinitol | 83.4 | HMDB0034219 |
| 645 | 23.865 | 221.1026 | Piceatannol | 71.3 | HMDB0004215 |
| 649 | 23.996 | 154.0933 | Guanosine | 68.2 | HMDB0000133 |
| 650 | 24.016 | 103.07 | L-sorbose | 94.7 | HMDB0001266 |
| 651 | 24.037 | 204.122 | Methyl beta-d-glucopyranoside | 98.3 | HMDB0029965 |
| 656 | 24.112 | 319.19 | Gluconolactone | 97.3 | HMDB0000150 |
| 661 | 24.272 | 129.1 | Melezitose | 77.7 | HMDB0011730 |
| 665 | 24.308 | 204.1255 | Galactose | 99.5 | HMDB0000143 |
| 666 | 24.31 | 204.13 | Maltotriose | 88.2 | HMDB0001262 |
| 667 | 24.316 | 146.0972 | Lanosterol | 62.9 | HMDB0001251 |
| 670 | 24.444 | 174.1233 | Cadaverine | 88.9 | HMDB0002322 |
| 671 | 24.505 | 319.2038 | Glucose | 96.9 | HMDB0000516 |
| 674 | 24.534 | 156.13 | Lactitol | 58.5 | HMDB0040937 |
| 675 | 24.547 | 174.1243 | L-lysine | 85.6 | HMDB0000182 |
| 678 | 24.681 | 132.087 | Montanic acid | 71.2 | HMDB0002348 |
| 680 | 24.728 | 299.233 | Pentacosanoic acid | 86.2 | HMDB0002361 |
| 683 | 24.779 | 357.1467 | 2,3-dihydrobutyraldehyde 1-phosphate | 79.7 |  |
| 684 | 24.8 | 147.07 | Mannose | 84.4 | HMDB0000169 |
| 692 | 24.978 | 299.2914 | 1-hexacosanol | 81.1 |  |
| 696 | 25.045 | 247.1733 | 2-monoolein | 69.1 | HMDB0011537 |
| 698 | 25.103 | 319.1833 | Sorbitol | 97.3 | HMDB0000247 |
| 705 | 25.412 | 217.13 | Methyl 6-deoxy-alpha-l-galactofuranoside | 86.9 |  |
| 708 | 25.526 | 217.137 | 1-kestose | 91.4 | HMDB0011729 |
| 709 | 25.542 | 466.311 | Bistris | 78 |  |
| 717 | 25.891 | 132.0865 | Lignoceric acid | 71.1 | HMDB0002003 |
| 718 | 25.895 | 378.1133 | Xanthurenic acid | 62.8 | HMDB0000881 |
| 719 | 25.972 | 95.09 | Chenodeoxycholic acid | 77.6 | HMDB0000518 |
| 724 | 26.085 | 129.08 | Cholest-4-en-3beta-ol | 94.6 |  |
| 726 | 26.105 | 204.128 | D-glucose | 99.5 | HMDB0000122 |
| 729 | 26.175 | 117.0433 | Palmitelaidic acid | 98.5 | HMDB0012328 |
| 736 | 26.47 | 333.1714 | Gluconic acid | 98 | HMDB0000625 |
| 738 | 26.499 | 147.08 | Isohexonic acid | 83.9 |  |
| 740 | 26.572 | 117.05 | Palmitic acid | 99.4 | HMDB0000220 |
| 741 | 26.588 | 313.29 | Barbital | 73.5 |  |
| 745 | 26.745 | 174.1233 | Glycyl tyrosine | 79.4 | HMDB0028853 |
| 751 | 26.929 | 318.1833 | Scyllo-inositol | 96.7 | HMDB0006088 |
| 755 | 27.108 | 217.135 | Lactobionic acid | 79.8 |  |
| 759 | 27.174 | 129.08 | Cholesterol | 98.6 | HMDB0000067 |
| 761 | 27.38 | 109.095 | Squalene | 67 | HMDB0000256 |
| 766 | 27.684 | 134.0962 | 3-methoxybenzenepropanoic acid | 68 | HMDB0011751 |
| 767 | 27.703 | 134.1 | N-acetyl-d-glucosamine | 89.2 | HMDB0000215 |
| 781 | 28.158 | 173.0864 | N-acetylgalactosamine | 83.2 | HMDB0000212 |
| 783 | 28.22 | 327.289 | Heptadecanoic acid | 90.3 | HMDB0002259 |
| 784 | 28.306 | 357.1418 | Bisphenol a | 71.7 | HMDB0032133 |
| 786 | 28.384 | 315.13 | D-ribose 5-phosphate | 94.3 | HMDB0001548 |
| 787 | 28.401 | 327.3368 | Octadecanol | 97.1 | HMDB0002350 |
| 790 | 28.566 | 357.1472 | D-ribulose 5-phosphate | 93.7 | HMDB0000618 |
| 793 | 28.709 | 319.1913 | Sedoheptulose | 96.6 | HMDB0003219 |
| 794 | 28.777 | 319.1871 | Allose | 96.1 | HMDB0001151 |
| 795 | 28.825 | 387.2765 | 2-deoxy-d-arabino-hexose 6-phosphate | 69.2 |  |
| 796 | 28.974 | 103.12 | 1-methylinosine | 60 | HMDB0002721 |
| 797 | 28.988 | 328.2919 | 2-deoxy-d-erythro-pentofuranose 5-phosphate | 72.1 |  |
| 801 | 29.218 | 117.05 | Erucic acid | 86.2 | HMDB0002068 |
| 802 | 29.218 | 117.0486 | Oleic acid | 82.3 | HMDB0000207 |
| 803 | 29.219 | 117.05 | Linoleic acid | 84.2 | HMDB0000673 |
| 804 | 29.223 | 202.1233 | L-tryptophan | 69.6 | HMDB0000929 |
| 814 | 29.554 | 117.0499 | Stearic acid | 79.9 | HMDB0000827 |
| 826 | 30.031 | 355.1081 | Epigallocatechin | 65.2 | HMDB0038361 |
| 830 | 30.285 | 315.1345 | Trehalose-6-phosphate | 79.8 | HMDB0001124 |
| 834 | 30.391 | 315.1383 | D-fructose-6-phosphate | 76.1 | HMDB0000124 |
| 849 | 30.785 | 387.1775 | D-fructose-1-phosphate | 78.8 | HMDB0001076 |
| 854 | 30.906 | 315.1388 | D-fructose-1,6-bisphosphate | 79.9 | HMDB0001058 |
| 856 | 30.944 | 314.2075 | 3'-adenylic acid | 58.5 | HMDB0003540 |
| 860 | 31.049 | 117.06 | Lithocholic acid | 75.5 | HMDB0000761 |
| 867 | 31.199 | 93.06778 | Beta-sitosterol | 77.6 | HMDB0000852 |
| 870 | 31.246 | 239.2059 | Dehydroabietic acid | 69.7 | HMDB0061925 |
| 875 | 31.369 | 204.1175 | Glucose 6-phosphate | 81.7 | HMDB0001401 |
| 880 | 31.448 | 367.3307 | Cis-gondoic acid | 77.6 | HMDB0002231 |
| 887 | 31.797 | 132.09 | Formononetin | 68.2 | HMDB0005808 |
| 890 | 31.94 | 318.176 | D-myo-inositol 4-phosphate | 82.3 | HMDB0001313 |
| 893 | 31.969 | 217.1352 | Uridine | 80.9 | HMDB0000296 |
| 901 | 32.241 | 179.0883 | Isochlorogenic acid | 57.6 |  |
| 905 | 32.361 | 356.3522 | Terbutaline | 62 | HMDB0015009 |
| 910 | 32.504 | 354.39 | Xanthine | 58.4 | HMDB0000292 |
| 916 | 32.79 | 91.07 | Docosahexaenoic acid | 97.4 | HMDB0002183 |
| 918 | 32.853 | 218.1396 | 2-monopalmitin | 77.8 | HMDB0011533 |
| 920 | 32.873 | 129.08 | Stigmasterol | 77.3 | HMDB0000937 |
| 925 | 33.128 | 371.3323 | 1-monopalmitin | 74.9 | HMDB0011564 |
| 930 | 33.27 | 307.2534 | Zeranol | 53.5 | HMDB0032702 |
| 942 | 33.768 | 205.1263 | Desyrel | 63.2 | HMDB0014794 |
| 957 | 34.213 | 218.1419 | 2-monostearin | 69.2 | HMDB0011535 |
| 958 | 34.254 | 204.1117 | Trisaccharide | 84.2 |  |
| 960 | 34.276 | 129.075 | 1-monoolein | 81 | HMDB0011567 |
| 964 | 34.415 | 399.3661 | 1-monostearin | 77 | HMDB0011131 |
| 989 | 35.594 | 193.1087 | Beta-tocopherol | 56.7 | HMDB0006335 |
| 990 | 35.67 | 223.1236 | Gamma-tocopherol | 75 | HMDB0001492 |
| 994 | 36.112 | 311.3028 | 4-aminophenol | 57.8 | HMDB0001169 |

Average Rt(min): mean retention time; Quant mass: Quantitative ion fragments; Total score: Total similarity score

Table S2. Post hoc power analysis of the subgroup comparisons and candidate biomarkers.

| Comparison | n (cases/HC) | Minimum detectable d (80% power, α = 0.05) | Candidate biomarker | AUC (95% CI) | Estimated achieved power vs AUC = 0.5 |
| --- | --- | --- | --- | --- | --- |
| P vs H | 12/8 | 1.35 | Hydroxylamine | 0.8021 (0.5920-1.000) | ~0.60 |
| A vs H | 18/8 | 1.25 | N-acetylputrescine | 0.9722 (0.9179-1.000) | >0.90 |
| U vs H | 7/8 | 1.53 | Glucose | 0.8929 | ~0.65 |
| U vs H | 7/8 | 1.53 | Phosphoenolpyruvate | 0.8750 | ~0.60 |
| U vs H | 7/8 | 1.53 | D-ribose | 0.8750 | ~0.60 |

P: pulmonary infection; A: abdominal infection; U: urinary tract infection; H/HC: healthy controls. d, Cohen's d. Power for AUC was estimated using the Hanley–McNeil variance approximation. Values are post hoc estimates based on the observed data and should be interpreted descriptively.
